# Bayesian Compressive Sensing of Sparse Signals with Unknown Clustering Patterns

**DOI:** 10.3390/e21030247

**Published:** 2019-03-05

**Authors:** Mohammad Shekaramiz, Todd K. Moon, Jacob H. Gunther

**Affiliations:** Electrical and Computer Engineering Department and Information Dynamics Laboratory, Utah State University, 4120 Old Main Hill, Logan, UT 84322-4120, USA

**Keywords:** compressed sensing (CS), sparse Bayesian learning (SBL), joint sparsity, cluster structured sparsity, single measurement vector (SMV), multiple measurement vectors (MMVs)

## Abstract

We consider the sparse recovery problem of signals with an unknown clustering pattern in the context of multiple measurement vectors (MMVs) using the compressive sensing (CS) technique. For many MMVs in practice, the solution matrix exhibits some sort of clustered sparsity pattern, or clumpy behavior, along each column, as well as joint sparsity across the columns. In this paper, we propose a new sparse Bayesian learning (SBL) method that incorporates a total variation-like prior as a measure of the overall clustering pattern in the solution. We further incorporate a parameter in this prior to account for the emphasis on the amount of clumpiness in the supports of the solution to improve the recovery performance of sparse signals with an unknown clustering pattern. This parameter does not exist in the other existing algorithms and is learned via our hierarchical SBL algorithm. While the proposed algorithm is constructed for the MMVs, it can also be applied to the single measurement vector (SMV) problems. Simulation results show the effectiveness of our algorithm compared to other algorithms for both SMV and MMVs.

## 1. Background and Introduction

**Figure 1:**
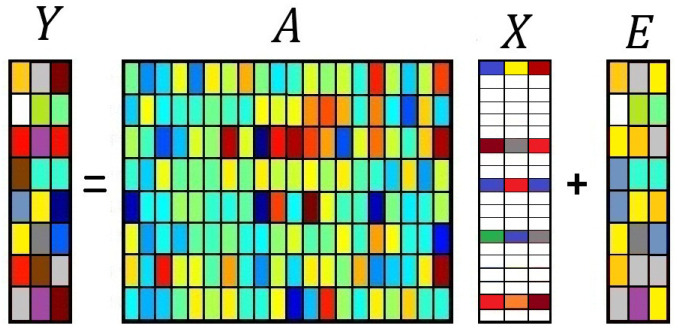
Example of the MMV structure.

Single and multiple measurement vector (SMV and MMV) problems are computational inverse problems in the compressive sensing (CS) area. CS provides the possibility of representing a sparse or compressible signal using a small set of non-adaptive linear measurements [1,2]. In linear CS, the *P*-dimensional signal $x\in R^{P}$ is modeled by the linear equation y=Φx, where $y\in R^{M}$ is the measurement vector (with M≪P) and $\Phi \in R^{M\times P}$ is a wide sensing matrix. The sensing matrix Φ is usually constructed from a Gaussian or Bernoulli random operator. In [3], it was shown that Φ also can be constructed from a class of circulant matrices based on deterministic sequences such as the Golay sequence [3]. In the CS context, it is further assumed that x is sparse under some proper basis Ψ, i.e., $x=\Psi x_{s}$, where $x_{s}$ denotes a sparse vector. A sparse vector contains few non-zero components. Combining the two above equations, we obtain $y\;=\;Ax_{s}$, where A=ΦΨ [4]. Since *A* is wide, the model is underdetermined, and CS looks for a sparse (if not the most sparse) solution ${\hat{x}}_{s}$ such that $y\;=\;A{\hat{x}}_{s}$ [5,6]. The SMV is a CS problem when *A* is known and the measurements are contaminated with noise e, i.e., $y\;=\;Ax_{s}+e$. The case where *Y* and $X_{s}$ are matrices is called the MMV problem, i.e., $Y\;\;=AX_{s}+E$. In the basic MMV model, it is assumed that all the columns of the solution matrix $X_{s}$ share joint sparsity, meaning that they have the same unknown non-zero locations. Figure 1 shows an example of the MMV structure.

The problem we address in this paper is for the recovery of sparse signals with an unknown clustering pattern via either SMV or MMVs. After providing an extensive survey of existing techniques in this area, we present a new hierarchical sparse Bayesian learning model and compare it to the existing algorithms. Though the formulation and modeling will be presented for the basic MMV, the proposed model is also applicable to SMV by simply considering vector cases rather than matrices in the model.

### 1.1. Literature Review on SMV and MMVs

Finding a sparse representation ${\hat{x}}_{s}$ in the SMV problem can be achieved using greedy algorithms such as matching pursuit (MP) and orthogonal matching pursuit (OMP) [5,7] or relaxed-to-be-convex methods (such as basis pursuit de-noising (BPDN) and the in-crowd algorithm) [8,9], the class of iterative shrinkage-thresholding algorithms (ISTA), and their variations such as fast iterative shrinkage-thresholding algorithm (FISTA), NESTA (a shorthand for Nesterov’s algorithm), and ISTA-NET [10,11,12], and sparse Bayesian learning (SBL) algorithms [13,14,15,16]. Similarly, there exist three main approaches for solving MMVs. The first approach is the extended version of the greedy-based SMV solvers such as MMV basic matching pursuit (M-BMP), MMV order recursive matching pursuit (M-ORMP), and MMV orthogonal matching pursuit (M-OMP) [17,18,19]. The second approach is relaxed-to-be-convex algorithms such as the joint $l_{2,0}$ approximation algorithm (JLZA) [20]. The third approach includes the SBL algorithms that are more flexible for incorporating prior knowledge on the structure of the solution compared to the greedy-based algorithms [21,22,23].

In some practical applications, the non-zero components of the sparse signal appear in clusters. For the MMV case, this means that in addition to the joint sparsity structure, the non-zeros also appear in clusters in each column of $X_{s}$ in $Y\;=\;AX_{s}+E$. This feature has been referred to as the clustered structure or block-sparsity pattern in the literature [7,24,25]. Applications of clustered sparsity for the SMV cases arise in problems such as gene expression analysis [26], image reconstruction of hand-written digits [27], and audio signals using the discrete cosine transform (DCT) basis [28]. Applications of MMVs can be found in neuromagnetic imaging [17], the reconstruction stage of Xampling (compressed-sensing of analog signals) for multi-band signals [7,24], and the direction of arrival (DOA) estimation problem [29]. For example, in magnetoencephalography (MEG), the goal is to investigate the locations where most brain activities are produced. The brain activities exhibit contiguity, meaning that they occur in localized regions [25]. Therefore, the measured signal at each snapshot can be modeled as a block-sparse SMV problem.

When taking successive and almost simultaneous snapshots from the phenomena, one expects the block-sparsity structure to be preserved. Hence, it is possible to model these activities with a block-sparse MMV problem where the block partitions are unknown a priori.

During the last decade, several greedy-based algorithms have been proposed to solve clustered pattern SMVs such as reduce MMV and boost (ReMBo) algorithm [7], block-OMP [30], structured OMP (StructOMP) algorithm [31], and group LASSO [32]. However, these algorithms need prior knowledge on the block sizes or the cluster pattern.

Bayesian learning models incorporate prior knowledge on the characteristics of the underlying signal. Starting with prior knowledge, these algorithms update their belief about the underlying features of interest in an unsupervised manner using the observations. Regarding the sparse recovery of SMV and MMVs, existing SBL algorithms can be mainly categorized into the two following approaches. The first and most common approach to impose sparsity on the solution is achieved by modeling each component of the solution with a zero-mean Gaussian prior accompanied with a Gamma distribution on the precision (inverse of variance) of the corresponding component [13,33,34,35]. In order to promote the clustering pattern, as well as sparsity in these models, different priors have been introduced on the variance of each component of the signal [15,28,36]. For example, in the case of clustered SMV using SBL with zero-mean Gaussian priors, Zhang and Rao incorporated the intra-block correlation structure (correlation structure in each block) [15]. In order to simplify the model, reduce the complexity, and suppress the over-fitting of the parameters in the model, they considered the same, but uncorrelated underlying covariance matrix for each possible block of the solution. The covariance matrix is updated via the expectation-maximization (EM) algorithm. In another work, Fang et al. used a zero-mean Gaussian prior where the precision on each component is statistically dependent on the precisions of the corresponding component and its two immediate neighbors [28].

The second SBL approach for the clustered sparse signal reconstruction uses a spike-and-slab prior [27,37,38,39,40]. These models have been applied to the SMV problem. Hernandez et al. proposed the generalized spike-and-slab prior, which is suitable for situations where prior information on the groups of components in the solution (that are expected to be jointly zero or jointly non-zero) is available [27]. Yu et al. made the spike and slab probabilities for each solution component depend on three possible patterns of the neighbor supports [37,40]. The patterns depend on whether the two immediate neighbors of each component are active, inactive, or only one of them is active. In [38], a Gaussian process prior was imposed on the spike-and-slab probabilities.

### 1.2. Idea Behind the Proposed Algorithm

In this paper, we present a new hierarchical Bayesian learning algorithm to solve the MMV problem for sparse signals with an unknown cluster pattern. We first establish a simple hierarchical Bayesian model for solving the general form of the MMV problem. In this initial model, we use the Bernoulli-Gaussian prior, which approximates the spike-and-slab prior, but in the sense that instead of employing spikes in the model, we have a binary vector that is to be learned to determine the supports of the sparse solution. Related algorithms can be found in [37,41,42]. In terms of Gaussian–Bernoulli modeling of the sparse signal, our initial model is close to the simplified form of [37,42], and in terms implementation, it uses the MCMC implementation with Gibbs sampler technique as in [37,41]. A binary matrix was used as a part of the Bayesian modeling in [41] for separating the foreground (sparse) component and the background (low-rank) component from the collection of noisy frames of a video recording. The difference between our initial model and [41] is that here, we use a binary vector to learn the supports of the solution for the *MMV problem with the joint sparsity structure*. However, this initial model only favors sparse solutions without any feature to promote the clustering pattern. The main reason for defining this initial model appears later in this paper where we modify the model not only to promote sparsity, but also to account for the clustered structure that may exist in the solution. The main contribution in this paper is related to the modified model, where we impose a prior based on the $l_{1}$-norm of the discrete gradient of the support vector s to promote clustering. This prior incorporates a parameter to account for the measure of contiguity, or “clumpiness,” in the supports of the solution. Assigning a large value to the hyperparameter represented by this parameter encourages the overall supports of the solution to have fewer on/off transitions, that is more contiguity of clustering in the supports. Similar to the other parameters, this parameter is also to be learned via our hierarchical SBL algorithm. Previously-developed algorithms do not have this control parameter for learning the pattern via the measure of overall clumpiness over the solution.

The proposed algorithm learns the supports and the corresponding values of the active components in the solution simultaneously. This task is accomplished by defining a Bernoulli-Gaussian-inverse Gamma distribution on the solution. Based on the built-in prior modeling, the solution tends to become sparse. In contrast, existing algorithms that use Gaussian-inverse Gamma prior modeling may need to perform some post-processing to remove the non-dominant components from the estimated solution. Existing algorithms in this area evaluate the performance based on the MSE reconstruction performance, but there are applications for which the support recovery is of equal or more importance. Examples can be found in compressive sampling and reconstruction of blind multi-narrowband signals in the continuous-to-finite (CTF) reconstruction stage, where the goal is to seek the edges of the sparsely-scattered narrowband signals to estimate the carrier frequencies [7,24]. In [7,24], the authors assumed that the number of narrowband signals is known, which yields the use of a modified OMP. The modified OMP is fed with the true sparsity level and the support block sizes of two. However, if the number of such signals is unknown, then we need an algorithm such as our proposed algorithm to learn the actual sparsity level, as well. For the problems raised in [7,24], it turns out that the supports of the solution in the CTF stage tend to clump together, which is representative of clustered pattern supports. For this problem, the supports of the solution are needed in order to figure out the locations of the actual carriers of the signals in the spectrum. Since our algorithm learns the support vector, it can naturally estimate the location of the carriers. In contrast, the other existing algorithms may tend to provide many supports, including non-dominant supports, which need to be post-processed to estimate the locations of the carriers. Another example is in [43], where the goal is to figure out the location of the sparsely-scattered multi-narrowband signals in the spectrum and then use this information to be able to fill out the empty spaces in the spectrum.

The novelty of our algorithm can be described as follows. Prior works in this area usually consider three hyperpriors commonly modeled by Gamma distributions, either on the precision of the Gaussian modeling on the solution or on the probabilities associated with Bernoulli modeling of the support vector elements, to promote clustered pattern solutions. These Gamma hyperpriors have different parameters to decide on each component of the solution based on the active/inactive status of its immediate components. By contrast, our model incorporates a total variation-like hyperprior, as a measure of the overall clustering pattern, on the support vector of the solution. Our model includes *one* control parameter in this prior to account for the emphasis on the amount of clumpiness in the support vector. This control parameter is learned in a Bayesian fashion, rather than having three different hyperpriors. Learning this parameter and the use of total variation-like prior on the support vector are novel.

Our proposed algorithm differs from [28] in two aspects. First, our model can be readily applied to either SMV or MMV problems, while the original PC-SBL algorithm proposed in [28] solves for the clustered pattern SMVs. Although it has been recently extended via generalized approximate message passing (GAMP) to solve for 2D problems [44], it needs some extra modifications to be used for the MMVs. Secondly, our model uses the Bernoulli-Gaussian prior, and it promotes the clustering pattern by adding hyperpriors on the supports of the solution, while in [28], this task is performed on the variances of the solution components. Yu et al. [37,40] used the spike-and-slab prior model and forced each mixing weight to depend on one of the three different possible active/inactive patterns of its immediate neighboring supports. This idea comes from the *k*-nearest neighbor approach in the clustering problems. Our approach differs from [37,40] due to the first reason we provided earlier for [28]. Tibshirani et al. presented an algorithm for SMVs referred to as the fused-lassoalgorithm, which promotes both sparsity and smoothness in the solution [26]. In this algorithm, the smoothness is promoted by using the absolute value of the difference between the estimated values of the successive components of the solution. In contrast, our proposed algorithm promotes sparsity and is able to learn the clustering pattern that may exist in the supports of the solution. The clustering pattern is learned by using the summed absolute value of the differences in the supports (our *sigma-delta* function), which distinguishes these algorithms. More specifically, we incorporate a total variation-like prior on the support vector of the solution rather than using such a prior on the solution vector itself. Finally, there are some SMV solvers that use the spike and slab prior model where the slab is modeled by a Gaussian scaled mixture model instead of just one Gaussian distribution [45]. It turns out that using this model can provide a better estimate of the underlying distribution of the non-zero elements. However, using this model increases the number of parameters to be learned even when we have only one mixing probability parameter to learn the supports. In [45], for the purpose of reducing the complexity of the algorithm, the expectation-maximization algorithm using approximate message passing was employed. Our measure of clumpiness can also be incorporated in this model to promote the clustering pattern.

Early development of this work was presented in [46], following which significant changes have been made. We have completely changed the update rule of some of the parameters, i.e., $\gamma_{p}$ in (15) and the controlling parameter α in (20) to learn the overall clumpiness of the supports. The convergence issue in the MCMC algorithm is addressed, and we explain how to track and monitor the convergence of the posterior distributions and make decisions on the supports based on the collected samples. Additionally, we compare the performance of our algorithm with other algorithms for both the SMV and MMVs, both on synthetic and real data.

This paper is organized as follows. In Section 2, we construct a basic hierarchical SBL model for solving the MMVs when the solution shares joint sparsity. Section 3 describes our main proposed algorithm, which extends this basic model to account for both joint sparsity and the unknown clustering pattern that may exist in the solution. In Section 4, we illustrate the performance of our proposed work compared to the other algorithms. Finally, Section 5 discusses the convergence diagnostic of the MCMC technique for the proposed algorithm. Section 6 presents conclusions.

## 2. Initial Model: Sparse Bayesian Learning for MMVs

As an initial model, here we construct a hierarchical SBL algorithm for the sparse recovery of basic MMVs with the joint sparsity structure that is expected to occur across the columns of the solution matrix. As discussed earlier, this model serves as the initial model, which we will modify in Section 3 for the clustered pattern sparse signals. We refer to this SBL algorithm as ordinary-SBL (O-SBL).

In this model, the supports of the solution are modeled by the binary vector s. Therefore, the sparse solution is described by s∘X, where s and *X* account for the support and the solution-values, respectively, and ∘ denotes element-by-element multiplication (Hadamard product) applied across the columns of *X*. The model for the MMV problem is:

(1) Y=A(s∘X)+E,

where $Y\;\in \;R^{M\times N}$, $A\;\in \;R^{M\times P}$, $s\;\in \;{\left\{0,1\right\}}^{P\times 1}$, $X\;\in \;R^{P\times N}$, and $E\;\in \;R^{M\times N}$. The matrix *Y* contains *N* columns of observed noisy data; *A* denotes the known sensing matrix; s is an unknown binary support-learning vector; *X* is an unknown solution-values matrix; and *E* represents the measurement noise. In the product s∘X, when N>1, the support vector s deals across the columns of *X*. The term s∘X is simply equivalent to diag{s}·X, where “·” is the regular matrix product and diag{·} creates a diagonal matrix from its argument vector.

**Figure 2:**
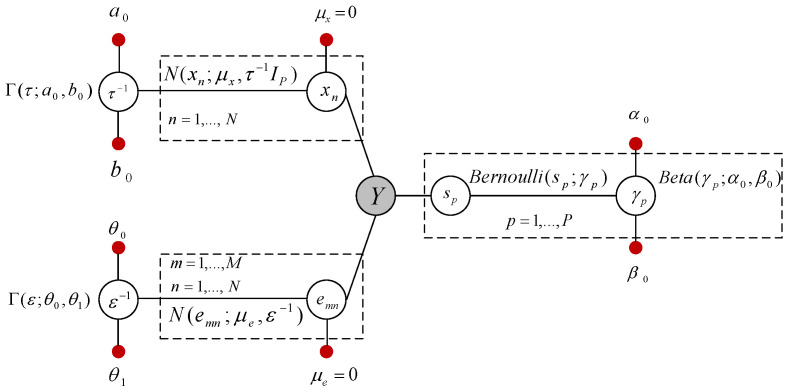
Graphical model of the Bayesian formulation (1).

A representation of a hierarchical Bayesian graphical model of the problem used in the development of our algorithm is portrayed in Figure 2.

The shaded node *Y* shows the observations, and the small solid nodes represent the hyperparameters. Each unshaded node denotes a random variable (or a group of random variables) [47]. The support-learning component s in (1) is a binary vector, and we model the elements of s as Bernoulli random variables, whose probabilities are governed by the prior $\gamma ={\left[\gamma_{1},\gamma_{2},\cdots ,\gamma_{P}\right]}^{T}$; that is,

(2) $$s_{p}\;∼\;\mathrm{Bernoulli}\left(\gamma_{p}\right),\;\gamma_{p}\;∼\;\mathrm{Beta}\left(\alpha_{0},\beta_{0}\right),p\;=\;1,\ldots ,P.$$

In order to favor the sparsity structure in s, on the basis of experimentation, we set $\alpha_{0}=\frac{1}{P}$ and $\beta_{0}=1\;-\;\alpha_{0}$, as suggested in [41].

The columns of the solution-value matrix $X=\left[x_{1},\ldots ,x_{N}\right]$ in (1) are assumed to be drawn *i.i.d.* according to the normal-gammadistribution:

$$x_{n}\;∼\;N\left(0,\tau^{-1}I_{P}\right),\tau \;∼\;\mathrm{Gamma}\left(a_{0},b_{0}\right),n\;=1\;,\ldots ,N,$$

where $a_{0}$ and $b_{0}$ denote the shape and rate of the Gamma distribution, respectively. For the purpose of reducing the model complexity, we use the same precision τ for all the components of *X*. Moreover, due to the lack of prior knowledge on the entries of *X*, we experimentally set the hyper-parameters to $a_{0}\;=\;b_{0}\;=\;10^{-3}$, endowing *X* *a priori* with a fairly high variance.

The entries of the noise component *E* are assumed to be drawn *i.i.d.* from a Gaussian distribution with the precision $\varepsilon^{-1}$. In our model, the precision $\varepsilon^{-1}$ is unknown and will be inferred, so that:

(3) $$e_{mn}∼N\left(0,\varepsilon^{-1}\right),\;m=1,\ldots ,M,\;n=1,\ldots ,N,\;\varepsilon ∼\mathrm{Gamma}\left(\theta_{0},\theta_{1}\right).$$

The hyper-parameters in (3) are set to $\theta_{0}=\theta_{1}=10^{-3}$. Referring to the graphical model in Figure 2, the joint probability distribution of the model can be written as:

(4) $$p\left(Y,s,X\right)\;\propto \;p\left(Y|s,X,\varepsilon \right)\left(\prod_{n=1}^{N}p\left(x_{n}|0,\tau^{-1}I_{P}\right)\right)p\left(s|\gamma \right)p\left(\gamma ;\alpha_{0},\beta_{0}\right)p\left(\tau ;a_{0},b_{0}\right)p\left(\varepsilon ;\theta_{0},\theta_{1}\right).$$

In the descriptions of the marginalized posterior distributions below, conditioning on −, as in $\left(s_{p}|-\right)$, is used to denote the inference on $s_{p}$ conditioning upon all relevant variables (including the observations).

- (5) $$\left(s_{p}|-\right)∼\mathrm{Bernoulli}\left(\frac{q_{1}}{q_{0}+q_{1}}\right),\;$$
  where $q_{1}=\gamma_{p}e^{-\frac{\varepsilon }{2}\left({∥a_{p}∥}_{2}^{2}\sum_{n=1}^{N}x_{pn}^{2}-2a_{p}^{T}\sum_{n=1}^{N}x_{pn}{\tilde{y}}_{n}^{-p}\right)}$ and $q_{0}=1-\gamma_{p}$.
  Here, ${\tilde{y}}_{n}^{-p}={\left[{\tilde{y}}_{1n}^{-p},\ldots ,{\tilde{y}}_{mn}^{-p}\right]}^{T}$, and the term ${\tilde{y}}_{mn}^{-p}$ is defined as:
  $${\tilde{y}}_{mn}^{-p}=y_{mn}-\sum_{l\neq p}^{P}a_{ml}s_{l}x_{ln}.$$
  The Appendix provides more details.
- $\left(\gamma_{p}|-\right)\propto p\left(s_{p}|\gamma_{p}\right)p\left(\gamma_{p};\alpha_{0},\beta_{0}\right)\propto \gamma_{p}^{s_{p}}{\left(1-\gamma_{p}\right)}^{1-s_{p}}\gamma_{p}^{\alpha_{0}-1}{\left(1-\gamma_{p}\right)}^{\beta_{0}-1}$
  Therefore, $\left(\gamma_{p}|-\right)\;∼\;\mathrm{Beta}\left(\alpha_{0}+s_{p}\;,\;\beta_{0}+1-s_{p}\right)$.
- $\left(x_{pn}|-\right)\;∼\;N\left(\mu_{pn},\sigma_{pn}\right)$, where $\mu_{pn}\;=\;\varepsilon s_{p}\sigma_{pn}a_{p}^{T}{\tilde{y}}_{n}^{-p}$ and $\sigma_{pn}\;=\;{\left(\tau \;+\;\varepsilon s_{p}^{2}{∥a_{p}∥}_{2}^{2}\right)}^{-1}$.
- $\left(\tau |-\right)\propto p\left(X|0,\tau^{-1}\right)p\left(\tau ;a_{0},b_{0}\right)\propto \tau^{\frac{NP}{2}}e^{-\frac{\tau }{2}{∥X∥}_{F}^{2}}\tau^{a_{0}-1}e^{-b_{0}\tau }$.
  Therefore, $\left(\tau |-\right)∼\mathrm{Gamma}\left(a_{0}+\frac{NP}{2},b_{0}+\frac{1}{2}{∥X∥}_{F}^{2}\right)$, where ${∥.∥}_{F}$ denotes the Frobenius norm. Finally,
- $\left(\varepsilon |-\right)\propto p\left(Y|s,X,\varepsilon \right)p\left(\varepsilon ;\theta_{0},\theta_{1}\right)\propto \varepsilon^{\frac{MN}{2}}e^{-\frac{1}{2}\varepsilon {∥Y-A\left(s\circ X\right)∥}_{F}^{2}}\varepsilon^{\theta_{0}-1}e^{-\varepsilon \theta_{1}}$.
  Thus, $\left(\varepsilon |-\right)\;∼\;\mathrm{Gamma}\left(\theta_{0}\;+\;\frac{MN}{2},\theta_{1}\;+\;\frac{1}{2}{∥Y\;-\;A(s\;\circ \;X)∥}_{F}^{2}\right)$.

In our implementation, we draw from the conditional posterior densities via Markov chain Monte Carlo (MCMC) using Gibbs sampling [41], as shown in the following pseudocode.

**O-SBL Algorithm**:
${\left\{\Theta^{\left(i\right)}\right\}}_{i=1}^{N_{\mathrm{collect}}}=O-\mathrm{SBL}\left(Y,A,\Theta_{0},N_{\mathrm{burn}-\mathrm{in}},N_{\mathrm{collect}}\right)$

**For**
$\mathrm{Iter}=1\;\mathrm{to}\;N_{\mathrm{burn}-\mathrm{in}}+N_{\mathrm{collect}}$
% Support-learning vector component  **For**
p=1toP   ${\tilde{y}}_{mn}^{-p}=y_{mn}-\sum_{l\neq p}^{P}a_{ml}s_{l}x_{ln}$, ∀m=1toM,∀n=1toN   $q_{0}=1-\gamma_{p}$   $q_{1}=\gamma_{p}e^{-\frac{\varepsilon }{2}\left({∥a_{p}∥}_{2}^{2}\sum_{n=1}^{N}x_{pn}^{2}-2a_{p}^{T}\sum_{n=1}^{N}x_{pn}{\tilde{y}}_{n}^{-p}\right)}$   $\left(s_{p}|-\right)∼\mathrm{Bernoulli}\left(\frac{q_{1}}{q_{0}+q_{1}}\right)$   % Solution-value matrix component   **For**
l=1toP    $\sigma_{x}={\left(\tau +\varepsilon s_{l}^{2}{∥a_{l}∥}_{2}^{2}\right)}^{-1}$    $\bar{\mu }=\varepsilon s_{l}\sigma_{x}a_{l}$    ${\tilde{y}}_{n}^{-l}=y_{n}-A\left(s\circ x_{n}\right)+s_{l}x_{ln}a_{l},\;\forall n=1\;\mathrm{to}\;N$    $\left(x_{ln}|-\right)∼N\left({\bar{\mu }}^{T}{\tilde{y}}_{n}^{-l},\sigma_{x}\right),\;\forall n=1\;\mathrm{to}\;N$   **End For**{l}   $\left(\gamma_{p}|-\right)∼\mathrm{Beta}\left(\alpha_{0}+s_{p}\;,\;\beta_{0}+1-s_{p}\right)$  **End For**{p}  $\left(\tau |-\right)∼\mathrm{Gamma}\left(a_{0}+\frac{NP}{2},b_{0}+\frac{1}{2}{∥X∥}_{F}^{2}\right)$  $\left(\varepsilon |-\right)∼\mathrm{Gamma}\left(\theta_{0}\;+\;\frac{MN}{2},\theta_{1}\;+\;\frac{1}{2}{∥Y\;-\;A(s\;\circ \;X)∥}_{F}^{2}\right)$  $\Theta^{\left(\mathrm{Iter}-N_{\mathrm{burn}-\mathrm{in}}\right)}\leftarrow \Theta$, $\forall \mathrm{Iter}>N_{\mathrm{burn}-\mathrm{in}}$**End For**{Iter}

In the above algorithm, $\Theta_{0}$ represents the set of initial values of the parameters of interest, drawn initially from the prior distributions defined in (2)–(3). We then run the O-SBL algorithm for the number of $N_{\mathrm{burn}-\mathrm{in}}$ iterations. Samples are not collected during the burn-in period. Then, $N_{\mathrm{collect}}$ more iterations are performed to collect the set of samples. For example, the estimate of the solution matrix *X* is computed from the sample mean, i.e., $\tilde{X}\;=\;1/N_{\mathrm{collect}}\sum_{n=N_{\mathrm{burn}-\mathrm{in}}+1}^{N_{\mathrm{burn}-\mathrm{in}}+N_{\mathrm{collect}}}{\hat{X}}^{\left[n\right]}$, where ${\hat{X}}^{\left[n\right]}$ denotes the collected samples for the solution matrix obtained from the corresponding approximated posterior distribution at the $n^{\mathrm{th}}$ iteration. As an alternative, one may use the samples obtained from the last iteration of the collection period as the estimate of the variable of interest. For example, $\tilde{X}\;=\;{\hat{X}}^{\left[N_{\mathrm{collect}}\right]}$. The MCMC convergence for the algorithm is discussed in Section 4.

## 3. Clustered Sparse Bayesian Learning

In this section, we modify the initial SBL model described in Section 2 to improve the support recovery performance of MMVs for the clustered sparse signals. We assume that the columns of the solution matrix are jointly sparse and that each of the vectors might have groups of clumps, i.e., groups of adjacent non-zero terms. Following [48], we measure the amount of clumpiness in the support-learning vector s by the absolute sum of the differences between successive elements of s,

(6) $$\Sigma \Delta \left(s\right)=\sum_{p=2}^{P}\left|s_{p}-s_{p-1}\right|.$$

**Figure 3:**
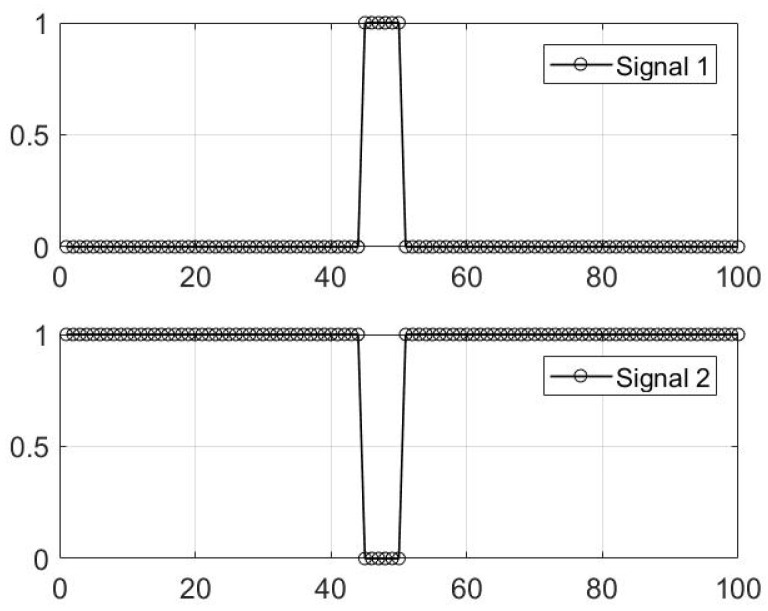
Example showing the effect of ΣΔ on the pattern of support vector s.

There exist fewer transitions in s, corresponding to a smaller ΣΔ, when the supports of the solution have a clustered pattern compared to an unstructured distribution of supports. The ΣΔ measure is used to establish a prior probability, which encourages clustered pattern supports by setting the prior for the support-learning vector s proportional to $e^{-\alpha (\Sigma \Delta )(s)}$ for some α>0. The parameter α specifies the significance of the clumpiness in the supports. Large values of α encourage more contiguity in the supports of s. However, the measure of ΣΔ (total variation on the support vector) itself is not sufficient to promote *sparse* clustered pattern supports, but rather only promotes clustered pattern solutions. Here, we provide a motivational example to clarify the reasoning for this. In Figure 3, we have two signals (one sparse and the other non-sparse), where both signals are of length 100.

According to Figure 3, both signals have the same measure of ΣΔ=2. As we can see in this figure, Signal 1 is sparse, while Signal 2 is non-sparse. Therefore, the measure of total variation is not sufficient to promote *sparse* clustered pattern solutions. Let us also investigate how the measure of total variation is affected by forcing a specific component in these two signals, active and inactive. The original active locations of Signal 1 are 45:50 (with MATLAB notation). Now, if we set the 44^th^ component in Signal 1 to become active, the measure of total variation does not change. Similarly, setting the 44^th^ component on Signal 2 to zero does not change the measure of total variation. This suggests that we need to further modify our model to promote sparsity, as well. For this purpose, we incorporate a binomial distribution into our prior model for s. This distribution contains the effect of the sparsifying hyperprior $\gamma_{p}$ and the sum over all the support components of the support vector (for both active and inactive status of $s_{p}$). Referring back to the example illustrated in Figure 3, for Signal 1, the sum over the supports of the signal (the number of active components) is six, while this measure is 94 for Signal 2. Therefore, the solution with the lower value of summation over the supports is sparser and of more interest.

We model the prior on the elements of the support-learning vector s as follows:

(7) $$\left(s_{p};\Omega_{p}\right)∼\mathrm{Bernoulli}\left(\Omega_{p}\right),\forall p=1,2,\ldots ,P,$$

where $\Omega_{p}\;:=\;\frac{\omega_{1,p}}{\omega_{0,p}+\omega_{1,p}}$ and:

(8) $$\omega_{k,p}=e^{-\alpha {\left(\Sigma \Delta \right)}_{k,p}}\mathrm{Binomial}\left(\Sigma_{k,p},P,\gamma_{p}\right),k\in \left\{0,1\right\},$$

and where $\Sigma_{k,p}$, k∈{0,1}, is defined as:

$$\Sigma_{k,p}\;:=\;k\;+\;\sum_{i\neq p}^{P}s_{p},$$

that is, it is the sum over all the elements of s for the case of forcing $s_{p}\;=\;k$. In other words, $\Sigma_{k,p}$ is the number of active elements in s when the $p^{\mathrm{th}}$ component of s is set to be one (active, via k=1) or zero (inactive, via k=0). For example, $\Sigma_{1,5}\;=\;1\;+\;\sum_{p\neq 5}^{P}s_{p}$. Furthermore, in (8), the term ${\left(\Sigma \Delta \right)}_{k,p}$ is the measure of clumpiness evaluated via (6) for the case of forcing the $p^{\mathrm{th}}$ component of s equal to *k*. This measures how the status of $s_{p}$ affects the contiguity over the supports of the solution. For example, ${\left(\Sigma \Delta \right)}_{0,5}\;=\;\sum_{p=2}^{P}\left|s_{p}-s_{p-1}\right|$, when we force $s_{5}\;=\;0$. This measures how the inactive status of $s_{5}$ affects the contiguity over the supports of the solution. The term $\Omega_{p}$ in the prior on the support learning vector s (7) can be further simplified into:

(9) $$\left(s_{p};\Omega_{p}\right)∼\mathrm{Bernoulli}\left(\Omega_{p}\right),\Omega_{p}\;:=\;\frac{1}{1+c_{p}e^{-\alpha {\left(\bar{\Sigma \Delta }\right)}_{p}}},\forall p,$$

where:

(10) $$c_{p}:=\frac{1-\gamma_{p}}{\gamma_{p}}\frac{\Sigma_{1,p}}{P-\Sigma_{0,p}},\forall p=1,\cdots ,P,$$

and:

(11) $${\left(\bar{\Sigma \Delta }\right)}_{p}\;=\;{\left(\Sigma \Delta \right)}_{0,p}\;-\;{\left(\Sigma \Delta \right)}_{1,p},\forall p=1,\cdots ,P.$$

Roughly speaking, this distribution favors drawing $s_{p}=1$ if this draw reduces ${\left(\Sigma \Delta \right)}_{1,p}$. Define ${\bar{c}}_{p}\;:=\;\left(1-\gamma_{p}\right)/\left(\gamma_{p}\right)$ and $\zeta_{p}\;:=\;\Sigma_{1,p}/\left(P-\Sigma_{0,p}\right)e^{-\alpha {\left(\bar{\Sigma \Delta }\right)}_{p}}$, and thus, $\Omega_{p}=1/\left(1+{\bar{c}}_{p}\zeta_{p}\right)$. If we set $\zeta_{p}\;=\;1$ in $\Omega_{p}$, then the prior on $s_{p}$ would be only governed by ${\bar{c}}_{p}$. In this case, $\Omega_{p}$ in (9) will be simplified into $\Omega_{p}\;=\;\gamma_{p}$, which is the same prior as we had earlier in (2). This prior only tends to promote sparsity without favoring the clustered pattern supports. By contrast, setting ${\bar{c}}_{p}\;=\;1$ in $\Omega_{p}$, for a sufficiently large value of α and ${\left(\bar{\Sigma \Delta }\right)}_{p}\;>\;0$, favors clustered pattern supports, which may lead to non-sparse solutions. Therefore, by incorporating both $c_{p}$ and the exponential term in the prior on $s_{p}$, defined in (9), the supports exhibit a trade off between sparsity and clustering.

**Figure 4:**
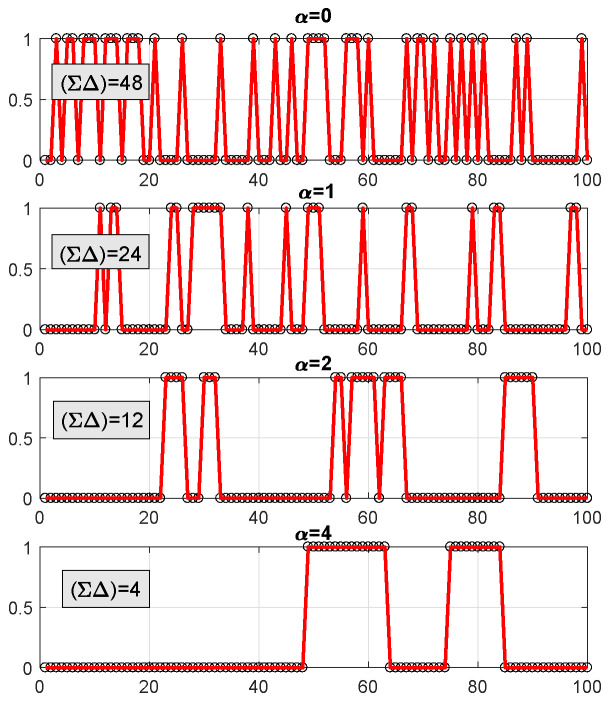
Example showing the effect of α on the pattern of support vector s.

In Figure 4, we demonstrate the effect of α defined in (8) and its role in the prior (9) on the support learning vector s. The figure shows draws of s according to (9) using (10) and (11).

Larger values of α result in lower ΣΔ(s), meaning that the support vector s will tend to have fewer transitions along its components, promoting clustering.

We employ a Gamma prior $\alpha \;∼\;\mathrm{Gamma}(a_{1},b_{1})$ on α, where $a_{1}$ and $b_{1}$ are hyperparameters denoting the shape and rate of the Gamma distribution, respectively. In order to promote the clustering pattern as a prior knowledge, we set $a_{1}\;=\;2\times 10^{-3}$ and $b_{1}\;=\;10^{-3}$, meaning that on average, we expect to have α=2 before incorporating the measurements. By setting these parameters to small values, as we have here, the learning process of α will not be biased by the prior, once the measurements are incorporated.

With these priors, the joint probability distribution for the complete model becomes:

(12) $$\begin{array}{c} p\left(Y,s,X\right)\;\propto \;p\left(Y|s,X,\varepsilon \right)\left(\prod_{n=1}^{N}p\left(x_{n}|0,\tau^{-1}I_{P}\right)\right)p\left(\tau ;a_{0},b_{0}\right)\times \\ \;p\left(\varepsilon ;\theta_{0},\theta_{1}\right)\left(\prod_{p=1}^{P}\left(s_{p}|\Omega_{p}\right)\right)p\left(\gamma |s,\alpha_{0},\beta_{0}\right)p\left(\alpha ;a_{1},b_{1}\right). \end{array}$$

**Figure 5:**
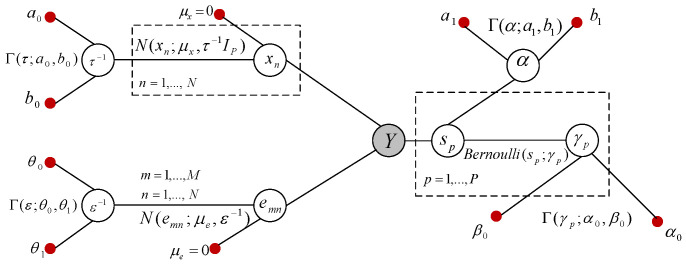
Graphical model of the Bayesian formulation (12).

The graphical model for the clustered pattern MMVs is shown in Figure 5.

Below, we describe the inference on the variables that are modified by (12) compared to (4). The inference on the other variables and parameters in the model is the same as those we described in Section 2.

- The posterior for $s_{p}$ is given by:
  $$\begin{array}{c} p\left(s_{p}|-\right)\;\propto \;p\left(Y|s_{p},X\right)p\left(s_{p}|\Omega_{p}\right)\;\;\propto \;\mathrm{Bernoulli}\left(s_{p}|\Omega_{p}\right)e^{-\frac{\varepsilon }{2}\left({∥a_{p}∥}_{2}^{2}\left(\sum_{n=1}^{N}x_{pn}^{2}\right)s_{p}^{2}-2a_{p}^{T}\left(\sum_{n=1}^{N}x_{pn}{\tilde{y}}_{n}^{-p}\right)s_{p}\right)}, \end{array}$$
  where $\Omega_{p}$ was defined in (7). Using the fact that $s_{p}$ is a binary random variable, the posterior inference on $s_{p}$ can be simplified to $\left(s_{p}|-\right)∼\mathrm{Bernoulli}\left(Q_{p}\right)$, where $Q_{p}=\frac{q_{1,p}}{q_{0,p}+q_{1,p}}$,
  $$q_{0,p}=\frac{1}{1+\left(\frac{\gamma_{p}}{1-\gamma_{p}}\right)\left(\frac{P-\Sigma_{0,p}}{\Sigma_{1,p}}\right)e^{-\alpha \left({\left(\Sigma \Delta \right)}_{1,p}-{\left(\Sigma \Delta \right)}_{0,p}\right)}},$$
  and:
  $$\begin{array}{c} q_{1,p}=\left(1\;-\;q_{0,p}\right)e^{\;-\;\frac{\varepsilon }{2}\left({∥a_{p}∥}_{2}^{2}\left(\sum_{n=1}^{N}x_{pn}^{2}\right)\;-\;2a_{p}^{T}\left(\sum_{n=1}^{N}x_{pn}{\tilde{y}}_{n}^{-p}\right)\right)}. \end{array}$$
  The posterior for $s_{p}$ can be further simplified into:
  (13) $$\left(s_{p}|-\right)∼\mathrm{Bernoulli}\left(\frac{1}{1+c_{p}\kappa_{p}e^{-\alpha {\left(\bar{\Sigma \Delta }\right)}_{p}}}\right),$$
  where $c_{p}$ and ${\left(\bar{\Sigma \Delta }\right)}_{p}$ were defined in (10) and (11), respectively, and:
  (14) $$\kappa_{p}:=e^{\frac{\varepsilon }{2}\left({∥a_{p}∥}_{2}^{2}\left(\sum_{n=1}^{N}x_{pn}^{2}\right)-2a_{p}^{T}\left(\sum_{n=1}^{N}x_{pn}{\tilde{y}}_{n}^{-p}\right)\right)}.$$
- The posterior for $\gamma_{p}$ is given by $p\left(\gamma_{p}|-\right)\;\propto \;p\left(\gamma_{p};\alpha_{0},\beta_{0}\right)\prod_{k=0}^{1}p\left(\omega_{k,p}|\Sigma_{k,p},\gamma_{p},\alpha ,s\right)$. Thus, $\left(\gamma_{p}|-\right)∼\mathrm{Beta}\left(\alpha_{1},\beta_{1}\right),$ where:
  (15) $$\begin{array}{c} \alpha_{1}:=\alpha_{0}\;+\;1\;+\;2\sum_{i\neq p}^{P}s_{i}\;,\;\beta_{1}:=\beta_{0}\;-\;1\;+\;2\left(P\;-\;\sum_{i\neq p}^{P}s_{i}\right). \end{array}$$
- The update rule for α is determined as follows. Using the joint probability distribution of the complete model (12) and discarding the terms that are unrelated to α, we have:
  (16) $$\begin{array}{cc} p(\alpha |-) & \;\propto \;p\left(\alpha ;a_{1},b_{1}\right)\prod_{p=1}^{P}p\left(s_{p}|\Omega_{p}\right)\\ & \;\propto \;\alpha^{a_{1}-1}e^{-b_{1}\alpha }\prod_{p=1}^{P}\mathrm{Bernoulli}\left(\frac{1}{1+c_{p}e^{-\alpha {\left(\bar{\Sigma \Delta }\right)}_{p}}}\right). \end{array}$$
  Due to the complicated nature of (16), we estimate α based on the current value of all the other variables and parameters of the model in the implemented MCMC approach. In other words, we compute:
  (17) $${\hat{\alpha }}_{MAP|Y,X^{\left[t\right]},s^{\left[t\right]},\Theta^{\left[t\right]}}^{\left[t+1\right]}=\arg\;\max_{\alpha }\;L_{\alpha |Y,X^{\left[t\right]},s^{\left[t\right]},\Theta^{\left[t\right]}},$$
  where $L_{\alpha }$ is obtained by taking the logarithm of (16) and is defined as:
  (18) $$\begin{array}{c} L_{\alpha }=\log\left\{p\left(\alpha ;a_{1},b_{1}\right)\prod_{p=1}^{P}p\left(s_{p}|\Omega_{p}\right)\right\}\\ \;\propto \;\left(a_{1}\;-\;1\right)\log\alpha -b_{1}\alpha \;+\;\sum_{p=1}^{P}\left\{s_{p}\log\Omega_{p}\;+\;\left(1\;-\;s_{p}\right)\log\left(1\;-\;\Omega_{p}\right)\right\}, \end{array}$$
  and may be further simplified into:
  (19) $$L_{\alpha }\propto \left(a_{1}\;-\;1\right)\log\alpha -b_{1}\alpha \;-\;\alpha \sum_{p=1}^{P}\left(1\;-\;s_{p}\right){\left(\bar{\Sigma \Delta }\right)}_{p}-\sum_{p=1}^{P}\log\left\{1\;+\;c_{p}e^{-\alpha {\left(\bar{\Sigma \Delta }\right)}_{p}}\right\}.$$
  Taking the derivative of $L_{\alpha }$ with respect to α and equating the resulting equation to zero, we obtain:
  $$\frac{a_{1}\;-\;1}{\alpha }\;-\;b_{1}\;-\;\sum_{p=1}^{P}\left(1\;-\;s_{p}\right){\left(\bar{\Sigma \Delta }\right)}_{p}\;+\;\sum_{p=1}^{P}\frac{{\left(\bar{\Sigma \Delta }\right)}_{p}}{1+\frac{1}{c_{p}}e^{\alpha {\left(\bar{\Sigma \Delta }\right)}_{p}}}=0.$$
  The maximum a *posteriori* point estimate of α conditioned on the measurements and the current estimate of hidden variables and the parameters of the model can be obtained by iteratively solving:
  (20) $$\frac{a_{1}\;-\;1}{\alpha^{\left[t+1\right]}}-b_{1}-\sum_{p=1}^{P}\left(1\;-\;s_{p}^{\left[t\right]}\right){\left(\bar{\Sigma \Delta }\right)}_{p}^{\left[t\right]}+\sum_{p=1}^{P}\frac{{\left(\bar{\Sigma \Delta }\right)}_{p}^{\left[t\right]}}{1\;+\;\frac{1}{c_{p}^{\left[t\right]}}e^{\alpha^{\left[t+1\right]}{\left(\bar{\Sigma \Delta }\right)}_{p}^{\left[t\right]}}}\;=\;0$$
  This update is computed at each iteration of the MCMC approach.

As an alternative approach, one can set α to a fixed predefined value. If under some prior knowledge, no clustering pattern is expected in the solution, one can set α to a small value close to zero. In case of expecting a highly clustered solution, we recommend setting α≫0.

**Remark** **1.** *In [46], the marginal posterior inference on $\gamma_{p}$ was estimated by $\left(\gamma_{p}|-\right)\;∼\;\mathrm{Beta}\left(\alpha_{0}\;+\;s_{p},\beta_{0}\;-\;s_{p}\right),\;\forall p\;=\;1,\cdots ,P.$ The idea behind the above update rule was the assumption of having a directed link from the node $\gamma_{p}$ to the node $s_{p}$ in Figure 5. However, in (15), we have removed this assumption and directly found the inference based on the relationship between the variables that we have in Figure 5. Furthermore, we have provided an analytical approach for the update rule of α, rather than the empirical approach discussed in [46].*

The pseudocode below describes our algorithm, referred to as C-SBL, which works for the clustered patterns SMV or MMVs.

**C-SBL Algorithm**:
${\left\{\Theta^{\left(i\right)}\right\}}_{i=1\;\mathrm{to}\;N_{\mathrm{collect}}}=C-\mathrm{SBL}\left(Y,A,\Theta_{0},N_{\mathrm{burn}-\mathrm{in}},N_{\mathrm{collect}}\right)$

**For**
$\mathrm{Iter}=1\;\mathrm{to}\;N_{\mathrm{burn}-\mathrm{in}}+N_{\mathrm{collect}}$
  % Support-learning vector component  **For**
p=1toP   ${\tilde{y}}_{mn}^{-p}=y_{mn}-\sum_{l\neq p}^{P}a_{ml}s_{l}x_{ln}$, ∀m=1toM,∀n=1toN   $c_{p}=\frac{1-\gamma_{p}}{\gamma_{p}}\frac{\Sigma_{1,p}}{P+1-\Sigma_{1,p}}$, ${\left(\bar{\Sigma \Delta }\right)}_{p}\;=\;{\left(\Sigma \Delta \right)}_{0,p}\;-\;{\left(\Sigma \Delta \right)}_{1,p}$   $k_{p}=e^{\frac{\varepsilon }{2}\left((∥a_{p}{∥}_{2}^{2}\sum_{n=1}^{N}x_{pn}^{2})-2a_{p}^{T}\left(\sum_{n=1}^{N}x_{pn}{\tilde{y}}_{n}^{-p}\right)\right)}$   $\left(s_{p}|-\right)∼\mathrm{Bernoulli}\left(\frac{1}{1+c_{p}k_{p}e^{-\alpha {\left(\bar{\Sigma \Delta }\right)}_{p}}}\right)$   % Solution-value matrix component   **For**
l=1toP    $\sigma_{x}={\left(\tau +\varepsilon s_{l}^{2}{∥a_{l}∥}_{2}^{2}\right)}^{-1}$    $\bar{\mu }=\varepsilon s_{l}\sigma_{x}a_{l}$    ${\tilde{y}}_{n}^{-l}=y_{n}-A\left(s\circ x_{n}\right)+s_{l}x_{ln}a_{l},\;\forall n=1,\cdots ,N$    $\left(x_{ln}|-\right)∼N\left({\bar{\mu }}^{T}{\tilde{y}}_{n}^{-l},\sigma_{x}\right),\;\forall n=1,\cdots ,N$   **End For**{*l*}   $\left(\gamma_{p}|-\right)∼\mathrm{Beta}\left(\alpha_{0}+1+2\sum_{k\neq p}^{P}s_{k}\;,\;\beta_{0}-1+2\left(P-\sum_{k\neq p}^{P}s_{k}\right)\right)$   **End For**{*p*}   $\left(\tau |-\right)∼\mathrm{Gamma}\left(a_{0}+\frac{NP}{2},b_{0}+\frac{1}{2}{∥X∥}_{F}^{2}\right)$   $\left(\varepsilon |-\right)∼\mathrm{Gamma}\left(\theta_{0}\;+\;\frac{MN}{2},\theta_{1}\;+\;\frac{1}{2}{∥Y\;-\;A(s\;\circ \;X)∥}_{F}^{2}\right)$   α: obtained from solving (20) for $\alpha^{\left[t+1\right]}$   $\Theta^{\left(\mathrm{Iter}-N_{\mathrm{burn}-\mathrm{in}}\right)}\leftarrow \Theta$, $\forall \mathrm{Iter}>N_{\mathrm{burn}-\mathrm{in}}$  **End For**{Iter}

Similar to the O-SBL algorithm, we perform MCMC inference in the implementation of the C-SBL using Gibbs sampling for all the variables and parameters of the model.

## 4. Simulation Results

Here, we compare the performance of our algorithm against other algorithms on both synthetic/simulated and real-world data for both the SMV and MMV problems.

### 4.1. Simulations on Synthetic Data

We first compare our proposed algorithm with other algorithms for the SMV problem, i.e., N=1 defined in (1). We then consider the MMV problem for the case where the number of columns in the solution matrix *X* is N=2,5.

#### 4.1.1. Performance for the SMV Problem

Each independently-generated trial is constructed as follows. We consider the solution-value vector $x\;\in \;R^{100}$, i.e., P=100 and N=1 in (1). The supports of the true solution are drawn randomly so that the support vector s exhibits a random clustered sparsity pattern. The total number of non-zeros in the sparse solution ($x_{s}=s\circ x$) is set to 25 for all the trials. The nonzero elements of x at the active supports of s are drawn *i.i.d.* from a zero-mean Gaussian distribution with variance $\sigma_{x}^{2}\;=\;1$. For each trial, the entries of the sensing matrix $A\;\in \;R^{M\times 100}$ are drawn *i.i.d.* from a zero-mean Gaussian distribution with variance one, and then, we normalize the columns of *A*. We vary the number of measurements *M* to show the performance as the ratio λ=M/P changes. The elements of the noise component are drawn *i.i.d.* from a Gaussian distribution $e_{m}\;∼\;N\left(0,\sigma^{2}\right)$. The SNR for all trials was 25 dB and is defined as SNR $=20\log_{10}\left(\sigma_{x}/\sigma \right)$. The measurement y is computed from $y\;=\;Ax_{s}+e$. The data described above were generated for 200 trials.

The recovery performance of the algorithms is demonstrated using both probability of support recovery and mean squared error. The probability of correct detection of a support location (probability of detection) $P_{D}$ and the probability of (erroneously) detecting a support location where there is none (probability of false alarm) $P_{FA}$ are respectively defined as $P_{D}=\left(\#\mathrm{Correct}\;\mathrm{detections}\right)/\left(\#\mathrm{Possible}\;\mathrm{correct}\;\mathrm{detections}\right)$, $P_{FA}\;=\;\left(\#\mathrm{False}\;\mathrm{detections}\right)\;/$ ((#Possibledetections)−(#Correctdetections)). A successful reconstruction is reported when all the supports of the true solution are recovered. The normalized mean-squared error is defined as:

(21) $$\mathrm{NMSE}\;\left(\mathrm{dB}\right):=20\log_{10}\frac{∥x_{s}-\hat{x_{s}}{∥}_{2}}{∥x_{s}{∥}_{2}},$$

where $\hat{x_{s}}$ is the estimated solution.

**Figure 6:**
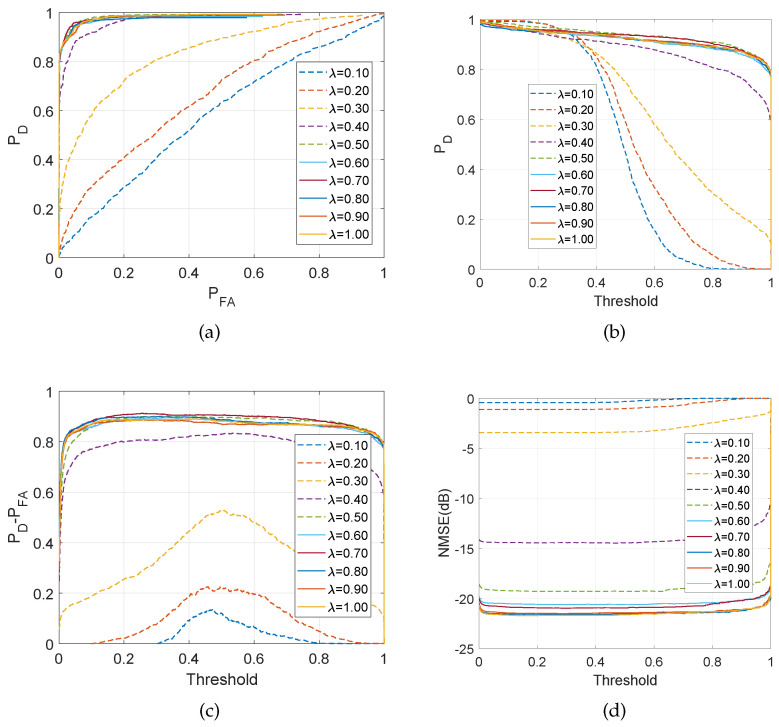
Aspects of performance of the proposed C-SBL algorithm for the SMV problem. (**a**) Empirical ROC; (**b**) Detection rate; (**c**) $P_{D}-P_{FA}$; (**d**) NMSE (dB).

Figure 6a–d demonstrates the aspects of the performance of the C-SBL algorithm. Figure 6a shows the performance of C-SBL using receiver operating characteristic (ROC) curves as the number of measurements, and equivalently the ratio λ=M/P varies. For λ>0.4 (M>40,P=100), C-SBL successfully finds all the supports of the true solution with generally a low false alarm rate. The algorithm exhibits high performance when the number of measurements is almost twice the number of true non-zeros in the solution (the true number of non-zeros was set to 25). In Figure 6b–d, we also illustrate the performance of C-SBL *vs.* the threshold, where the threshold is defined as follows. We average over all of the $N_{\mathrm{collect}}$ collected samples of the support learning vector, where each component belongs to {0,1}. In other words, we compute ${\hat{s}}_{ave}\;=\;1/N_{\mathrm{collect}}\sum_{n=N_{\mathrm{burn}-\mathrm{in}}+1}^{N_{\mathrm{collect}}}{\hat{s}}^{\left[n\right]}$. Then, those indices in the resulting vector ${\hat{s}}_{ave}$ that contain values greater than the threshold are chosen as the estimated supports of the solution (setting the threshold to 0.5 results in the sample mean of the collected samples). In Figure 6b, we illustrate the detection rate *vs.* the threshold as the ratio M/P changes (if we decide on the supports based on all the samples obtained in both burn-in and collected periods, then the detection rate would become zero for the threshold of one for all λ). Figure 6c shows the difference between detection rate and false alarm rate of C-SBL *vs.* the threshold. The higher values of $P_{D}\;-\;P_{FA}$ indicates the higher overall support recovery of the algorithm. In Figure 6c, there is a threshold of around 0.5, where $P_{D}-P_{FA}$ has a peak for λ≤0.4. For the case of λ>0.4, C-SBL reaches its highest performance with a wide range of threshold of approximately [0.1,0.9]. This verifies that estimating the support learning vector based on the sample mean (threshold of 0.5) provides a high performance for all λ. Finally, Figure 6d shows the C-SBL behavior in terms of normalized mean-squared error, defined in (21), *vs.* the threshold. In Figure 6d, the error term for each λ remains almost constant regardless of the threshold. This means that one can take a threshold that leads to a very high detection rate, even for a very low number of measurements, without any major change in terms of the error. However, according to Figure 6a and Figure 6c, different choices of the threshold result in different false alarm rates. According to Figure 6d, as the number of measurements becomes around twice the sparsity level (λ≥0.5), the error becomes almost negligible.

**Figure 7:**
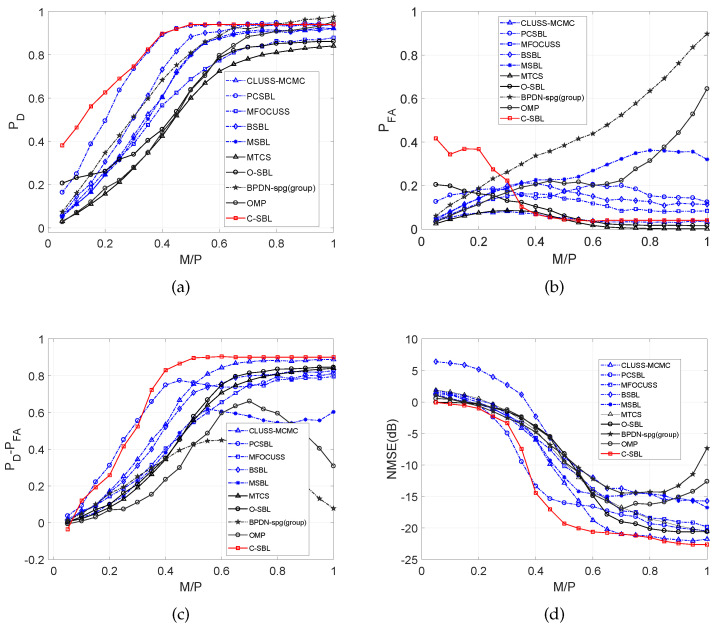
Comparisons of various algorithms in the SMV case. (**a**) Detection rate; (**b**) False alarm rate; (**c**) $P_{D}-P_{FA}$; (**d**) NMSE (dB).

We now compare the performance of C-SBL and O-SBL with other algorithms, specifically: CLUSS-MCMC algorithm for solving clustered structure compressive sensing using Markov chain Monte Carlo method [37], orthogonal matching pursuit (OMP) algorithm [5,49], MMV focal underdetermined system solver (MFOCUSS) [17], block-sparse Bayesian learning algorithm (BSBL) [15,23], MMV sparse Bayesian learning algorithm (MSBL) [22], basis pursuit denoising algorithm for group sparsity (BPDN-group) using the spectral projected gradient for $l_{1}$ minimization (SPGL1) solver [50], the single-task version of multi-task compressive sensing algorithm (MTCS) [34,51], and PC-SBL [28,52]. In all of the algorithms, we discarded those estimated elements in the solution with an amplitude less than 0.01 from the support set. In Figure 7a–d, the results for the O-SBL and C-SBL algorithms are based on the sample mean of the collected samples.

In Figure 7a, we demonstrate the empirical results of detection rate *vs.* the ratio M/P.

Figure 7a shows that C-SBL provides the best performance in terms of detecting the true supports of the solution. In Figure 7b, the false alarm rate in support recovery is illustrated, where we see that for M/P<0.35, our algorithm has a higher false alarm rate in support recovery at the cost of providing a higher detection rate within the same range for M/P. In contrast, the rates for C-SBL, O-SBL, CLUSS-MCMC, and MTCS become almost the same and have the lowest values.

Figure 7c compares the performance algorithms in terms of the trade off between the detection rate and false alarm rate in support recovery, in which C-SBL and PC-SBL show almost the same performance for M/P<0.35. However, C-SBL outperforms all the other algorithms for M/P>0.35. Figure 7d illustrates the comparison of NMSE between the true and estimated solution. We observe that C-SBL provides lower error among the other algorithms for a wide range of M/P.

**Remark** **2.** *The MATLAB codes for BSBL, MSBL, and MFOCUSS were obtained from [53]. For BSBL, the noise flag was set to two (small noise). and the block-size of h=2 was considered. For MSBL, we activated the option for learning the tuning parameter and initialized it by the true noise variance. For the MFOCUSS algorithm, the regularization parameter was set to $10^{-3}$. Based on some initial experiments, we decided to use the default settings for CLUSS-MCMC [54] and PCSBL [52]. The parameters of the Gamma prior on the noise variance for MTCS [51] were both set to one.*

**Figure 8:**
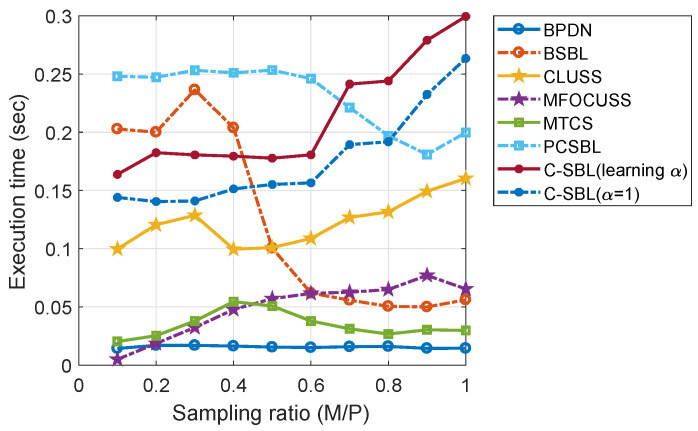
Execution-time comparison for the SMV case.

It has been very common in the literature to demonstrate the performance primarily on NMSE, so that successful recovery is reported when the NMSE becomes lower than some pre-defined value. In that sense and by referring to Figure 7d, we see that our algorithm provides the lowest error rate for a wide range of M/P. In addition, our algorithm demonstrates good performance on the ROC plot, showing high detection against the false alarm rate. Finally, in Figure 8, we show the average run-time comparison of the respective algorithms for 500 randomly-generated SMVs in the same way stated earlier. In Figure 8, the legend C-SBL(learning α) denotes the execution time of the C-SBL algorithm for the case where the algorithm learns α from (20), while in C-SBL(α=1), we do not use (20) and instead experimentally set α=1.

According to Figure 8, for low sampling ratios, which is of more interest in CS problems, the lowest execution times belong to THE PBDN, MFOCUSS, MTCS, and CLUSS-MCMC algorithms, respectively. However, as we showed in Figure 7, these algorithms do not provide good accuracy in signal reconstruction compared to the other algorithms. Furthermore, these algorithms do not account for learning the unknown clustering pattern. Comparing the execution time of the PC-SBL, B-SBL, and C-SBL algorithms for low sampling ratios, we observe that C-SBL demonstrates reasonably low execution time. Furthermore, notice that the C-SBL and CLUSS-MCMC algorithms are the only algorithms that are implemented using the MCMC method, and based on Figure 8, we observe that they both show the same behavior with respect to the change of the sampling ratio. The reason for the higher execution time of C-SBL against CLUSS-MCMC is again due to the extra computations that C-SBL requires to account for the clustering pattern.

#### 4.1.2. Performance for the MMV Problem with N=2

We first demonstrate the performance of C-SBL in terms of detection rate and false alarm rate in support recovery as the ratio M/P and NMSE.

**Figure 9:**
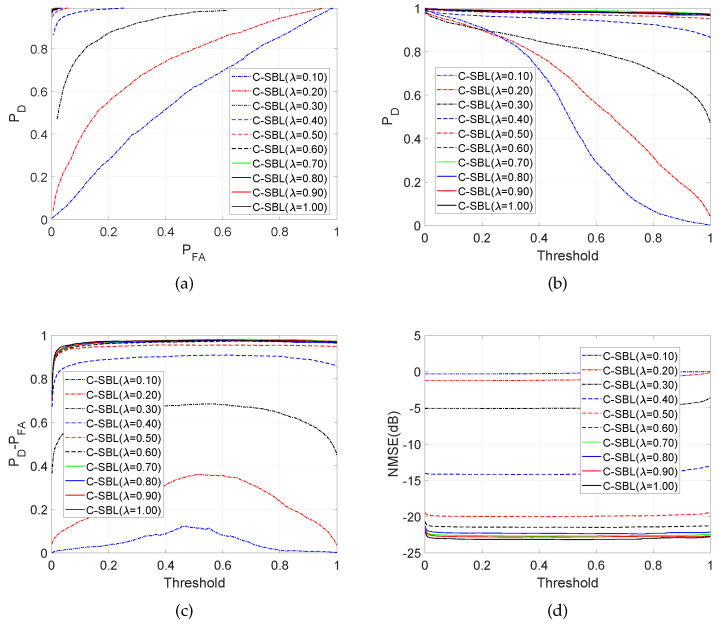
Aspects of the performance of C-SBL for MMV (with N=2). (**a**) Empirical ROC; (**b**) Detection rate; (**c**) $P_{D}-P_{FA}$; (**d**) NMSE (dB).

Figure 9a displays ROC curves. Comparing the results demonstrated in Figure 6a with Figure 9a, increasing the number of columns in the solution from N=1 to N=2 provides considerable improvement in the support recovery. Figure 9b illustrates the detection rate of C-SBL for the MMVs (with N=2) *vs.* different threshold values. Once M/P≥0.4 (over 40% compression and the sparsity of 25), almost full success in support recovery is attained regardless of the selected threshold value. The difference between the detection rate and false alarm rate of C-SBL *vs.* the threshold is shown in Figure 9c. There is a threshold of around 0.5, where $P_{D}\;-\;P_{FA}$ has a peak for λ≤0.4. For λ>0.4 in the MMV case, C-SBL reaches its highest performance almost regardless of the chosen threshold value. Therefore, we make a decision on the supports based on computing the sample mean, i.e., setting the threshold equal to 0.5. Figure 9d shows the NMSE *vs.* the threshold for the clustered MMV problem using the C-SBL algorithm.

**Figure 10:**
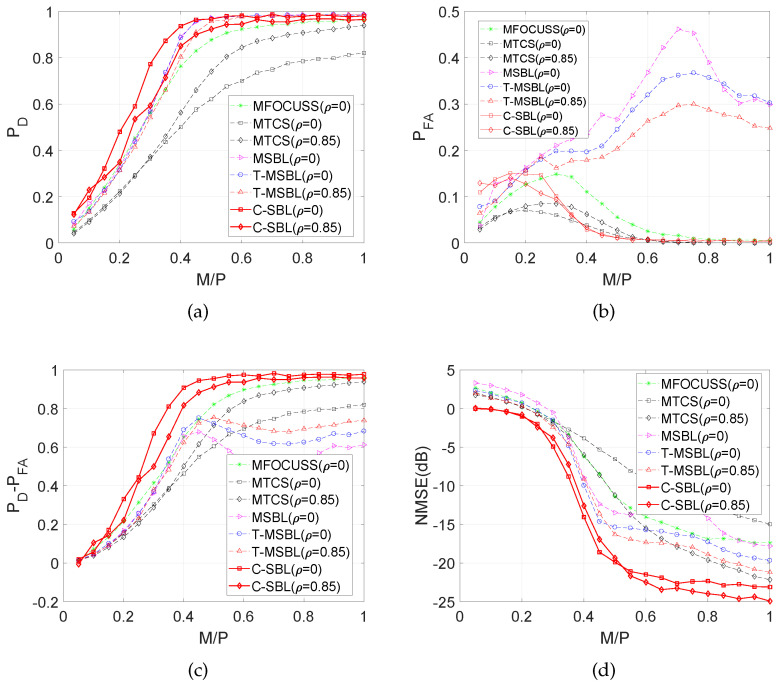
Comparison of various algorithms in the MMV case with N=2. (**a**) Detection rate; (**b**) False alarm rate; (**c**) $P_{D}-P_{FA}$; (**d**) NMSE (dB).

Finally, Figure 10a–d compares the performance of C-SBL against the other algorithms. We compare the results of C-SBL with the following algorithms: MFOCUSS [17], MSBL [22], T-MSBL [55,56], and MTCS [34]. Notice that T-MSBL is devised for correlated signals, while our model does not account for this feature. Although MTCS does not promote the clustering pattern, we use it as a baseline for our comparisons. We consider two sets of simulations. Our setup for the simulations is similar to the SMV case, as was described earlier. The only difference is in generating the true solution matrices. In the first case, we generate uncorrelated columns for the solution matrices. In the legend of Figure 10a–d, the uncorrelated cases are denoted by ρ=0. In the second case, the columns of the solution matrix for each trial are correlated with the correlation factor of ρ=0.85.

Figure 10a illustrates the detection rate in support recovery for clustered pattern MMVs (with N=2), in which we observe that C-SBL has the highest performance among the other algorithms for the uncorrelated case. Furthermore, we observe that C-SBL competes with T-MSBL for the correlated case. In Figure 10b, it is clear that C-SBL provides a lower false alarm rate in terms of support recovery compared to the MSBL and T-MSBL algorithms. The best performance belongs to MTCS, but it provided the lowest performance in terms of detection rate, as was shown in Figure 10a. For overall comparison, Figure 10c shows the simulation results in terms of the difference between the detection rate and false alarm rate in support recovery when varying the ratio M/P. According to Figure 10c, the overall performance of C-SBL is higher than the other algorithms. The NMSE comparisons are illustrated in Figure 10d, where we see that C-SBL provided the lowest error. According to Figure 10a–d, C-SBL is more successful than the compared algorithms in terms of both support recovery and estimating the non-zero values of the true solution.

#### 4.1.3. Performance for the MMV Problem with N=5

**Figure 11:**
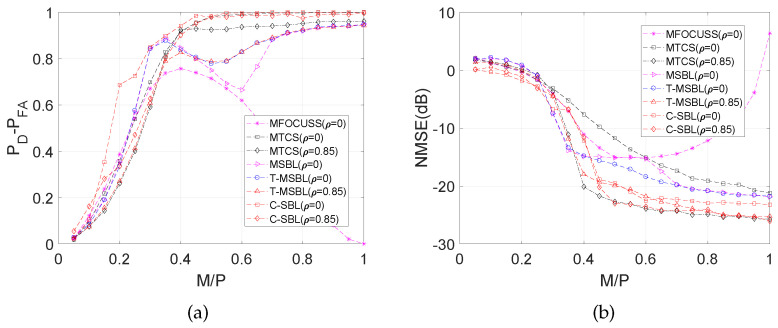
Comparison of various algorithms in the MMV case with N=5. (**a**) $P_{D}-P_{FA}$; (**b**) NMSE (dB).

Here, we perform simulations on synthetically-generated data in the same way explained previously except setting N=5. The burn-in and collection periods of C-SBL were set to 2000 and 1000, respectively. Figure 11 illustrates the performance comparison results for both the uncorrelated case (ρ=0) and the correlated case (with ρ=0.85).

According to Figure 11a, the best performance in terms of the difference between the detection rate and false alarm rate belongs to the C-SBL algorithm. The lowest performance belongs to FOCUSS, where as the sampling ratio becomes greater than 0.4, FOCUSS starts to activate more components in s. As a result, the false alarm rate increases, and this yields the smaller $P_{D}\;-\;P_{FA}$. For a sampling ratio of one, all the components of s are active, resulting in $P_{D}\;-\;P_{FA}\;=\;0$. The performance of FOCUSS would be still acceptable if the NMSE for high sampling ratios would have became very low, meaning that the wrongly-determined supports had very low amplitudes. However, according to the error curve of FOCUSS in Figure 11b, this does not happen, meaning that MFOCUSS crashed for moderately high and high sampling ratios. For sampling ratios λ>0.5, the best performance in terms of $P_{D}-P_{FA}$ belongs to both C-SBL and MTCS. Notice that they were both able to provide almost full support recovery. However, for low sampling ratios like λ≤0.4, the best support recovery belongs to C-SBL. For example, for λ=0.2, the C-SBL provided $P_{D}\;-\;P_{FA}$ of around 0.7, while the other algorithms provided values less than 0.4. This should justify the merit of the C-SBL algorithm. In summary, C-SBL demonstrates the best performance in support recovery for the uncorrelated case.

Figure 11b compares the error between the true and estimated solution. C-SBL provides the lowest error for the most possible range of λ (λ∈[0.05,0.25)∪[0.45,1]) for the uncorrelated case.

#### 4.1.4. Interpretation of the results for the correlated case

For the correlated case, Figure 11a shows that C-SBL performed a little bit better than the two other compared algorithms in terms of support recovery, even though the C-SBL model does not account for the correlation that may exist across the columns of *X*. According to Figure 11b, MTCS, T-MSBL, and C-SBL have almost the same overall performance in terms of error.

### 4.2. Experiments on Real Data

In this section, we compare the performance of C-SBL against other algorithms on real data. Specifically, we consider the image reconstruction problem for the SMV case using the MNISTdataset. For the comparisons of the MMV case, the problem of blind sub-Nyquist sampling and reconstruction of multi-narrowband signals is considered.

#### Performance for the SMV Case (Experiments on MNIST Data)

Here, we evaluate the performance of the algorithms in reconstructing images of hand-written digits, using the well-known MNIST dataset [57]. MNIST consists of 70,000 gray-scale images of 28×28 hand-written digits. Experiments are conducted on a randomly-chosen set of hand-written digits from “0”–“9” from this dataset. Due to space considerations, we show only some of the results. The original images are upsampled to size 100×100 pixels, and the pixel values were normalized to be within [0,1]. Then, the pixel values were subtracted from one, and those with a value of less than 0.3 were set to zero, similar to the binary pixel values in [58,59]. The threshold of 0.3 was obtained based on the average threshold of all the images using Otsu thresholding (the actual average threshold was 0.268). The corresponding matrix of pixel values of each image is then treated as the true sparse signal of interest, denoted by the true solution matrix *X*.

**Figure 12:**
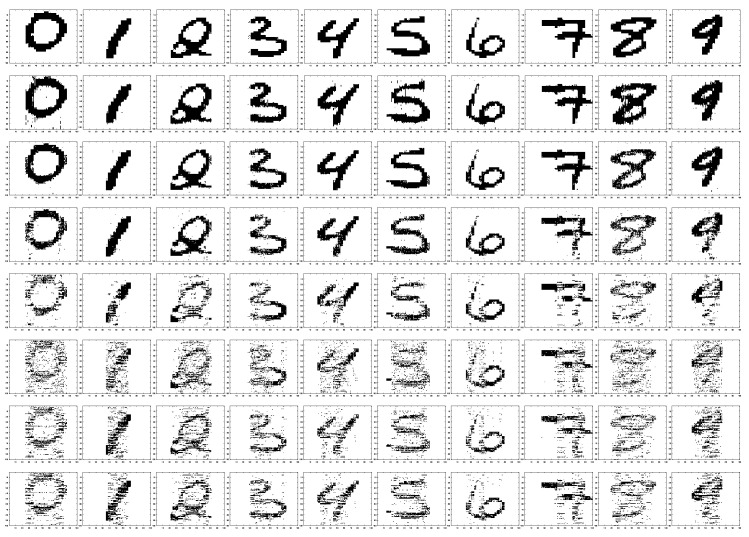
Results of reconstructed images for the SMV case. The first row illustrates the non-zero locations of the true hand-written digits. The other rows from top to bottom show the performance in terms of supports using the C-SBL, BSBL (h = 4), PCSBL, CLUSS-MCMC, MTCS, MFOCUSS, and BPDN algorithms, respectively.

For the SMV case, we solve each column of *X* for each digit one at a time. The number of measurements for each column of *X* is set to 55 and $x_{n}\in R^{100},\forall n=1,\cdots ,100$, i.e., we consider a compression of 55% measurements for each vector $x_{n}$. We randomly generated the sensing matrix *A* in the same way we described earlier. Then, each column of the matrix *A* is normalized to have a unit norm. The hand-written images of MNIST are already naturally sparse since most pixels in these images are inactive, i.e., they have a small number of non-zero pixels. The measurements for each column of the digits are computed by $y_{n}=Ax_{n}+e_{n}$ with SNR = 25 dB, where e is a Gaussian noise accounting for the measurement noise. This setting follows some of the other recent work in this area [60,61,62,63,64]. We feed all the algorithms with the measurement vector y and the same sensing matrix *A*. The generated measurement noise matrix is the same for the digits. Once ${\hat{x}}_{n},\forall n=1,\cdots ,100$ is known, we collect the results and then stack them all together and reconstruct the digits. For the purpose of demonstrating the support recovery performance, in Figure 12, we illustrate how successful the algorithms were in finding the non-zero pixel locations. Since in the compared algorithms, except our proposed C-SBL, the models do not have the support-leaning vector s, we performed the following. In the reconstruction, we set the estimated pixel values less than 0.3 to zero, the same way as we treated the actual images. This means that we zeroed out the brightest pixels with the normalized value of lower than the threshold 0.3 and set the non-zero survival pixel values to one. However, since the proposed C-SBL algorithm already can provide the estimates on the active locations via s, the thresholding process is not required. The first column of images in Figure 12 shows the true hand-written digits, and the other columns show the results of processing with other algorithms.

We compare the performance of the C-SBL algorithm against other algorithms in the reconstruction of the images via both the support and pattern recovery. In Table 1, we evaluate the reconstruction based on the difference between the detection rate and false alarm rate ($P_{D}-P_{FA}$) in terms of support recovery. In Table 2, the performance is evaluated based on the success in pattern recovery. For this purpose, we stack all the columns of the true matrix *X* for each digit into a single column. We then construct the corresponding support vector, where the index of pixels with non-zero value will be replaced by “1” in the corresponding support vector. The true measure of clumpiness for each digit will then be computed via (6). We do the same procedure for computing the estimated measure of clumpiness in the reconstructed digits and provide the results in Table 2. In Table 3, the error between the true and the reconstructed images is represented.

According to the results shown in Table 1, the best results in support recovery belong to the C-SBL algorithm. Furthermore, Table 2 shows that C-SBL was more successful in learning the underlying clustering pattern of the digits. In terms of the reconstruction error, Table 3 shows that the C-SBL and BSBL algorithms compete with each other, with some results being better for C-SBL and some being better for BSBL.

### 4.3. Performance for the MMV Case (Experiments on Blind Multi-Narrowband Signal Sampling and Reconstruction

**Figure 13:**
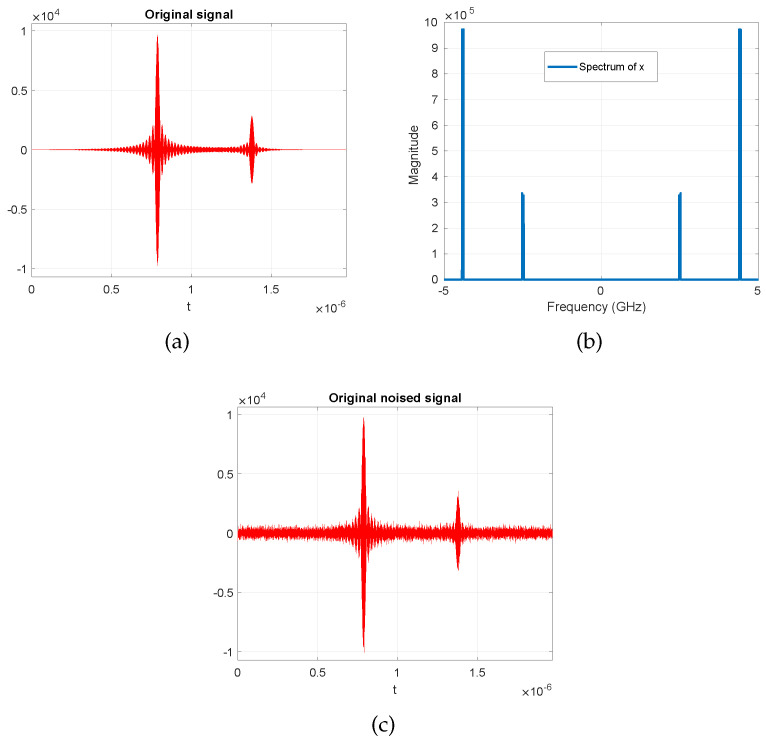
An example of a multi-narrowband signal for comparison purposes.(**a**) Original signal; (**b**) Spectrum of the signal; (**c**) Original noisy signal.

**Figure 14:**
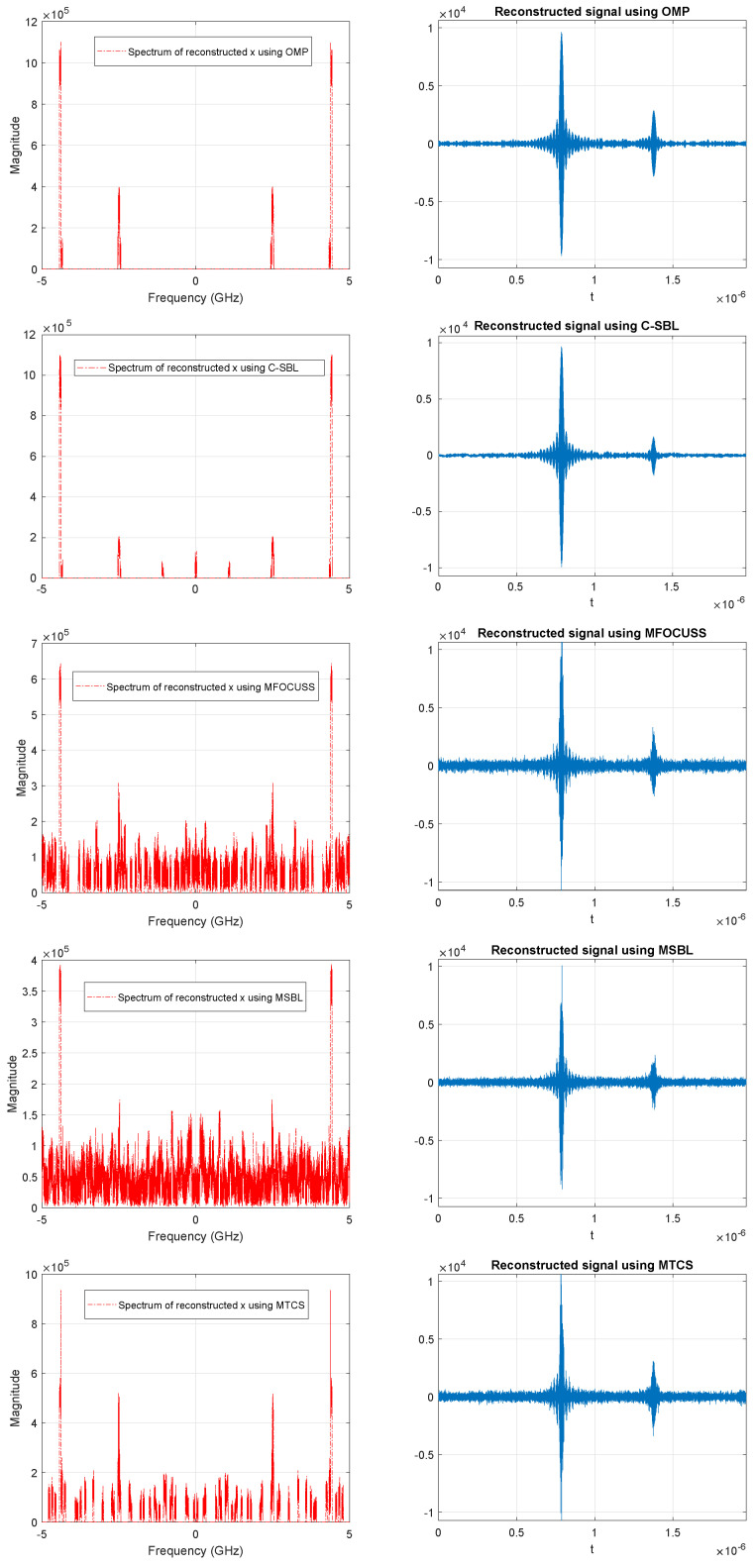
Results of estimated spectrum of the signal and the reconstructed signal in the CTF stage of Xampling when using the OMP, C-SBL, MFOCUSS, MSBL, and MTCS algorithms.

In this section, we consider the problem of blind sub-Nyquist sampling and reconstruction of multi-narrowband signals. The notion of blindness here means that the frequency support is unknown, and it occupies only a small portion of a wide spectrum [65]. In the original problem, it is assumed that the number of sparsely-scattered bands and their bandwidth are *known*, while the carrier locations are unknown at the receiver. The sub-Nyquist sampling in [65] is performed in the modulated wideband converter (MWC) stage, which multiplies the analog signal by a bank of $M_{ch}$ periodic waveforms followed by low-pass filtering and then sampling the outputs uniformly at a low rate far less than the Nyquist rate. The periodic waveforms intentionally alias the spectrum such that a portion of each band appears in the baseband. The technique used here is referred to as Xampling for the compressive sensing of analog signals [24,65]. The MMV problem appears in the reconstruction stage of the Xampling framework, referred to as the continuous-to-finite (CTF) stage. In this stage, the low rate samples, obtained from the MWC stage, are fed to the CTF stage to estimate the support vector. The estimation problem here involves solving for the sparse solution of an underdetermined system of linear equations, which has the structure of the MMV problem. The active bands (spectrum slices) of the signal are reconstructed based on the estimated supports. For more detail, refer to [24,65,66,67]. In our example, the signal of interest is a multi-band signal containing two pairs of bands, i.e., $N_{0}\;=\;4$, where each band is of width B=50 MHz, and it is assumed that the Nyquist rate of the multi-band signal is as high as $f_{Nyq}\;=\;10$ GHz. The true carriers are set to $f_{c_{1}}\;=\;2.4956$ MHz and $f_{c_{2}}\;=\;4.4086$ MHz, which are assumed to be unknown for the simulation purposes. The number of channels are set to $M_{ch}\;=\;70$, and the energies of the bands are set to $E_{1}\;=\;1$ and $E_{2}\;=\;2$, respectively. All the other settings of the signal model and the sampling parameters are defined the same as the implementation used in [68,69], which do not appear here due to the space consideration. In the simulations, the MMV problem that all algorithms need to solve is of the form Y=AX, where P=195, M=70, and N=8. The actual sparsity level is k=8, which here we assume to be unknown. Having no information on *k* here means that we assume that the number of active bands in the signal is also unknown. In Figure 13, we illustrate an example of a multi-narrowband signal, its spectrum, and the signal when contaminated with noise, which is used for the simulation purposes. Furthermore, Figure 14 shows the comparison of the OMP, our proposed algorithm (C-SBL), MFOCUSS, MSBL, and MTCS in estimating the spectrum of the signal to reconstruct the signal. Notice that *the OMP algorithm is fed with the actual sparsity level (k=8)*, while this information is assumed *unknown* to the other algorithms. The results shown for the OMP serve as a baseline in our comparisons, but the OMP requires more information to solve the problem, so the comparison is not exactly fair.

According to the results obtained in Figure 14, the C-SBL algorithm performed better than the alternatives in estimating the spectrum supports (closest support recovery to the OMP), which resulted in better signal reconstruction. Again, the OMP in these simulations is fed with the true sparsity level with the true support block sizes.

In Table 4, the reconstruction error in terms of NMSE (dB) is represented. According to the results, we see that the C-SBL algorithm outperformed the alternatives in reconstructing the signal.

The reason that the alternative algorithms did not provide low reconstruction error is due to the fact that their estimated spectrum supports of the signal became non-sparse, as illustrated in Figure 14.

## 5. Convergence Diagnostics of the MCMC Implementation

**Figure 15:**
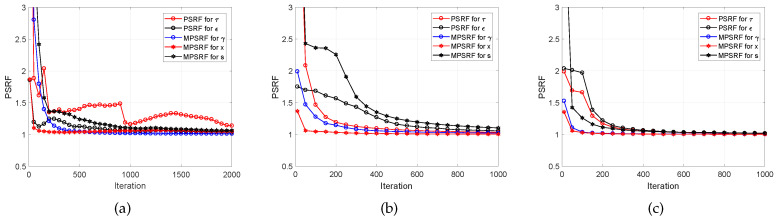
Examples showing the evolution of PSRF for the precision on the solution τ and the measurement noise precision ε and MPSRF for the solution vector x, the support vector s, and the mixing-coefficient vector γ for sampling ratios of 0.2, 0.5, and 0.9. (**a**) Example with M= 20; (**b**) Example with M= 50; (**c**) Example with M= 90.

Convergence is an important issue in order to determine the burn-in period of MCMC algorithms [37]. There is work on the convergence of iterative simulations and their inference using multiple sequences via potential scale reduction factor (PSRF) and multiple-PSRF (MPSRF) in [70,71]. For example, in [37], the convergence issue of the CLUSS-MCMC algorithm was resolved via studying the evolution of MPSRF in the collected samples for the sparse signal and its corresponding variances and the measure of PSRF for the noise variance. Following the same approach, in Figure 15, we provide some examples demonstrating the evolution of the PSRF for 20 independent chains of our proposed C-SBL algorithm. In these examples, we generated random clustered pattern sparse signals of length N=100, a sparsity level of 25, and with the number of measurements M= 20, 50, and 90.

**Figure 16:**
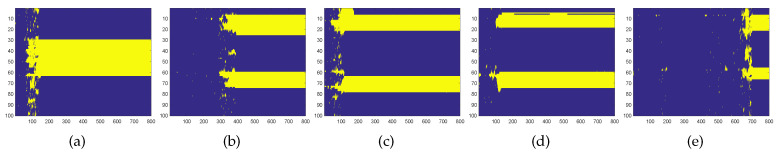
Examples showing samples of the support learning vector s. The vertical axis shows the elements of s, and the horizontal axis represents the iterations. (**a**) Example 1; (**b**) Example 2; (**c**) Example 3; (**d**) Example 4; (**e**) Example 5.

In Figure 15a, we demonstrate the PSRF and MPSRF for the variables and parameters of interest in our model for an example with the low sampling ratio of 0.2. According to the plot, the Gibbs sampler converges very quickly for ε, γ, and x, i.e., the convergence measure of PSRF became close to one with few iterations. However, the convergence of the distribution on s is slower. The convergence of the precision on *X* was the slowest. According to Gelman’s discussion in [72], one may prefer to set the burn-in period based on the PSRF close to 1.2. Based on this criterion, we can set the burn-in period to approximately 2000 iterations for the example made in Figure 15a. In Figure 15b, we provide another example for the case with the moderate sampling ratio of 0.5. As can be seen in this plot, the distributions of all the variables and parameters of interest converged faster than when the sampling ratio was 0.2. For this example, a burn-in period of around 600 is satisfactory. Figure 15c illustrates another example for the high sampling ratio of 0.9. In this plot, we observe that a burn-in period of around 200 suffices. Since in Figure 7 and Figure 9, Figure 10 and Figure 11, we wanted to show the average of the overall performance of our algorithm, we first performed the following and then set the burn-in period based on the obtained experimental results. For each sampling ratio, we generated 100 random trials for solving the SMV and MMV problems. In these trials, we assessed the average PSRF measure for all the variables and parameters of interest based on Gelman’s criterion in [72]. We monitored the average number of elapsed iterations until the variations on the outcomes of the estimated s became negligible for a fair number of iterations (this is easy to monitor since the outcome of the posterior on $s_{p}$ is Bernoulli). This is equivalent to monitoring the trace plots, as suggested by Neal [72]. More specifically, we monitored the posterior distribution on the support learning vector s, using (5) for O-SBL and (13) for C-SBL, based on the samples obtained from MCMC implementation. In Figure 16, we illustrate some examples of support learning vector s using the C-SBL algorithm with the number of measurements M=55. Each plot shows the learning process of $s\;\in R^{100}$, represented by the samples drawn from (13), as a function of the number of iterations. Using the experimental results based on both the PSRF evaluation and monitoring the outcomes of s, we then set fixed burn-in periods for the simulations illustrated in Section 4. In other words, since we wanted show the average performance, we preferred not to assess the PSRF of each simulated example, but rather using a fixed experimental burn-in period. Below, we provide the details on the burn-in and the collection periods of both the C-SBL and O-SBL algorithms for the simulation results illustrated in Section 4. In simulations on the synthetic data and the MNIST for the MMVs, we set the burn-in period to 500 followed by 500 iterations for the collection period. The same settings were used for the SMV case on the MNIST. In the experiments on synthetic data for the SMV, we set $N_{\mathrm{burn}-\mathrm{in}}\;=\;2000$ followed by $N_{\mathrm{collect}}\;=\;1000$ iterations for the collection period for the sampling ratios of M/P≤0.4. For M/P>0.4, we set $N_{\mathrm{burn}-\mathrm{in}}\;=\;1000$ and $N_{\mathrm{collect}}\;=\;1000$. Thus, it might be the case that the burn-in period is required to be more than what we set. The effect of the need for a longer burn-in period can be observed in the results of Figure 7, Figure 10, and Figure 11 for low sampling ratios. The convergence diagnostic and the effect of burn-in period can also be detected in Figure 6 and Figure 9. It should be clear from Figure 6c that for sampling ratios over 0.4, the average detection performance is almost independent of the threshold. The performance reveals that the approximated posterior distribution on s has already been stabilized. However, we see a different behavior for lower sampling ratios in Figure 6c. The posterior distribution on s is Bernoulli, and the variations on the supports in the iterative samples directly affect the performance in the support recovery. We see in Figure 6c that the average sample mean of the collected samples occurred around the threshold of 0.5. This can be interpreted as follows. The burn-in period may have been required to be larger than our setting, but the posterior distributions have been almost stabilized. Therefore, even for a lower burn-in period and sufficient iterations for the collection period, we could still extract the information required for estimating the supports via computing the sample mean of the collected samples.

Computing the MSPRF for s needed some modifications. The estimated posterior variance of s is assessed based on the mixture of all the simulated sequences divided by the average of the variances within each sequence [72]. The main issue with the MPSRF for s occurred in our simulations when computing $\hat{R}$. In fact, this matrix became ill-conditioned, and the issue was with the fact that sequences on s were either zero or one. We dealt with this issue by adding random draws from a zero-mean Gaussian with the variance of $10^{-8}$ to the samples of s and then measured the MPSRF.

## 6. Conclusions

The O-SBL algorithm simultaneously learns both the supports and solution-value matrix for the MMVs with the joint sparsity structure. As the main contribution of this paper, the method was then extended to account for the case where the solution also exhibits an unknown clustered sparsity pattern. For this purpose, we introduced the C-SBL algorithm, which incorporates a total variation-based prior on the supports of the solution to learn the underlying clustered pattern. Based on simulations, we observed that C-SBL provides competitive performance for both the SMV and MMVs compared to the other algorithms.

Although C-SBL provides encouraging results, the MCMC implementation is computationally expensive. In future work, we will consider alternative approaches to MCMC implementation such as the variational Bayes inference technique [40,73].

## Figures and Tables

**Table 1:** SMV case: comparing the reconstruction performance in terms of $P_{D}-P_{FA}$ for the digits. The bold face numbers show the best results in terms of support recovery.

Algorithm	Digit 0	Digit 1	Digit 2	Digit 3	Digit 4	Digit 5	Digit 6	Digit 7	Digit 8	Digit 9
C-SBL	**0.9530**	**0.9954**	**0.9592**	**0.9643**	**0.9834**	**0.9690**	**0.9847**	**0.9890**	**0.9250**	**0.9794**
BSBL [15,23,53]	0.9204	0.9819	0.9341	0.8301	0.9617	0.9479	0.9116	0.9568	0.7469	0.9263
PCSBL [28,52]	0.7622	0.9544	0.8717	0.7281	0.8961	0.8270	0.8468	0.7787	0.5746	0.7046
CLUSS [37,54]	0.4265	0.6689	0.4803	0.4981	0.6421	0.5878	0.7179	0.6098	0.3123	0.4233
MTCS [34,51]	0.3030	0.4828	0.3380	0.4102	0.4779	0.4074	0.5989	0.5042	0.2740	0.3620
MFOCUSS [17,53]	0.3652	0.6510	0.4012	0.4378	0.5197	0.4961	0.5734	0.5054	0.2997	0.4324
BPDN [50]	0.3701	0.6360	0.4502	0.4161	0.5212	0.4967	0.5859	0.5370	0.3251	0.4052

**Table 2:** SMV case: comparing the performance in terms of learning the clustering pattern via the measure of clumpinesss (ΣΔ) for the true and the reconstructed digits. The bold face numbers show the best results in terms of pattern recovery of the supports of the solution.

Algorithm	Digit 0	Digit 1	Digit 2	Digit 3	Digit 4	Digit 5	Digit 6	Digit 7	Digit 8	Digit 9
True value	208	80	290	306	166	316	220	240	358	168
C-SBL	**244**	**78**	**266**	**284**	**176**	**324**	**196**	**241**	**340**	**172**
BSBL [15,23,53]	320	84	402	458	200	344	262	276	750	228
PCSBL [28,52]	634	126	528	636	346	632	344	532	1080	560
CLUSS [37,54]	1018	370	960	758	570	804	446	664	1152	708
MTCS [34,51]	1774	1022	1742	1200	1194	1496	760	1178	1646	1298
MFOCUSS [17,53]	1532	878	1508	1082	936	1220	684	1124	1346	1186
BPDN [50]	1478	818	1440	1054	908	1192	646	1014	1352	1172

**Table 3:** SMV case: comparing the performance in terms of the reconstruction error NMSE (dB) for the reconstructed digits. The bold face numbers show the best results in terms of reconstruction error.

Algorithm	Digit 0	Digit 1	Digit 2	Digit 3	Digit 4	Digit 5	Digit 6	Digit 7	Digit 8	Digit 9
C-SBL	−8.0264	**−17.0256**	−9.2213	**−7.3822**	**−13.9387**	−10.1113	**−13.9837**	−9.6984	−3.7184	−8.3104
BSBL [15,23,53]	**−11.6553**	−16.4167	**−12.0378**	−7.2124	−13.5679	**−12.7994**	−11.3422	**−13.3647**	**−5.4846**	**−11.3947**
PCSBL [28,52]	−5.3168	−13.1082	−8.2997	−4.9542	−8.8138	−6.8723	−8.3111	−5.3168	−2.9960	−4.1409
CLUSS [37,54]	−1.5934	−4.1480	−2.1561	−2.6419	−4.1116	−3.3908	−6.1695	−3.1560	−0.9962	−1.3608
MTCS [34,51]	0.4987	−0.3990	0.1748	−0.4367	−0.9529	−0.1357	−2.6530	−0.8175	0.6790	0.4506
MFOCUSS [17,53]	−1.1638	−3.1979	−1.3832	−1.7897	−2.4664	−2.2302	−3.4462	−2.2340	−0.7605	−1.4593
BPDN [50]	−1.2096	−3.0473	−1.7805	−1.6362	−2.5115	−2.2342	−3.6447	−2.5400	−1.0469	−1.3351

**Table 4:** Comparing the reconstruction performance in terms of NMSE (dB) for the blind multi-narrowband signal. The two bold face numbers show the best results, which belong to OMP and C-SBL.

Algorithm	OMP [5,49]	C-SBL	MTCS [34,51]	MFOCUSS [17,53]	MSBL [22,53]
NMSE (dB)	**−17.5898**	**−14.2124**	−3.3095	−3.2028	−1.7223

## Appendix A

Below we describe the derivation of the inference equation for $s_{p}$ provided in (5). According to the joint probability distribution (4), we have,

(A1) $$\left(s_{p}|-\right)\propto p\left(Y|s_{p},X,\varepsilon \right)p\left(s_{p}|\gamma_{p}\right),\;\mathrm{and}\;\left(s_{p}|\gamma_{p}\right)∼\mathrm{Bernoulli}\left(\gamma_{p}\right),\;\forall p=1,\cdots ,P,$$

and:

(A2) $$\begin{array}{c} \log p(Y|s,X,\varepsilon )\propto -\frac{\varepsilon }{2}({\left(y_{11}-\sum_{p=1}^{P}a_{1p}s_{p}x_{p1}\right)}^{T}\left(⋆\right)+\cdots +\\ {\left(y_{M1}\;-\;\;\;\sum_{p=1}^{P}\;a_{Mp}s_{p}x_{x_{p1}}\right)}^{T}\left(\;⋆\;\right)\;+\;\cdots \;+\;{\left(y_{MN}\;-\;\;\sum_{p=1}^{P}\;\;a_{Mp}s_{p}x_{pN}\right)}^{T}\left(\;⋆\;\right)), \end{array}$$

where “⋆” in ${\left(\kappa \right)}^{T}\left(⋆\right)$ denotes “κ”. Define ${\tilde{y}}_{mn}^{-p}=y_{mn}-\sum_{l\neq p}^{P}a_{ml}s_{l}x_{ln},\forall \left\{m,n,p\right\}$, and substitute into (A2) to obtain:

(A3) $$\begin{array}{cc} \log p(Y|s_{p},X,\varepsilon ) & \propto -\frac{\varepsilon }{2}\left(s_{p}^{2}{∥a_{p}∥}_{2}^{2}\sum_{n=1}^{N}x_{pn}^{2}-2s_{p}\left(x_{p1}\sum_{m=1}^{M}a_{mp}{\tilde{y}}_{m1}^{-p}+\cdots +x_{pN}\sum_{m=1}^{M}a_{mp}{\tilde{y}}_{mN}^{-p}\right)\right)\\ & \propto -\frac{\varepsilon }{2}\left(s_{p}^{2}{∥a_{p}∥}_{2}^{2}\sum_{n=1}^{N}x_{pn}^{2}-2s_{p}\left(a_{p}^{T}\sum_{n=1}^{N}x_{pn}{\tilde{y}}_{n}^{-p}\right)\right), \end{array}$$

where ${\tilde{y}}_{n}^{-p}\;:=\;{\left[{\tilde{y}}_{1n}^{-p},\cdots ,{\tilde{y}}_{Mn}^{-p}\right]}^{T}$. Using (A1) and (A3), we have:

$$\left(s_{p}|-\right)\;\propto \;\gamma_{p}^{s_{p}}{\left(1\;-\;\gamma_{p}\right)}^{s_{p}}e^{-\frac{\varepsilon }{2}\left(s_{p}^{2}{∥a_{p}∥}_{2}^{2}\sum_{n=1}^{N}x_{pn}^{2}\;-\;2s_{p}a_{p}^{T}\sum_{n=1}^{N}x_{pn}{\tilde{y}}_{n}^{-p}\right)}.$$

Since $s_{p}\;\in \;\left\{0,1\right\}$, the marginalized posterior inference on this parameter can be simplified into (5).
